# MicroRNA let-7 and viral infections: focus on mechanisms of action

**DOI:** 10.1186/s11658-022-00317-9

**Published:** 2022-02-14

**Authors:** Arash Letafati, Sajad Najafi, Mehran Mottahedi, Mohammad Karimzadeh, Ali Shahini, Setareh Garousi, Mohammad Abbasi-Kolli, Javid Sadri Nahand, Seyed Saeed Tamehri Zadeh, Michael R. Hamblin, Neda Rahimian, Mohammad Taghizadieh, Hamed Mirzaei

**Affiliations:** 1 Department of Virology, School of Public Health, Tehran University of Medical Sciences, Tehran, Iran; 2 Department of Biotechnology, School of Advanced Technologies in Medicine, Shahid Beheshti University of Medical Sciences, Tehran, Iran; 3 Faculty of Medicine, Mashhad University of Medical Sciences, Mashhad, Iran; 4 Department of Virology, Faculty of Medicine, Iran University of Medical Sciences, Tehran, Iran; 5 Department of Medical Genetics, Faculty of Medical Sciences, Tarbiat Modares University, Tehran, Iran; 6 Infectious and Tropical Diseases Research Center, Tabriz University of Medical Sciences, Tabriz, Iran; 7 School of Medicine, Tehran University of Medical Sciences, Tehran, Iran; 8 Laser Research Centre, Faculty of Health Science, University of Johannesburg, Doornfontein, 2028 South Africa; 9 Endocrine Research Center, Institute of Endocrinology and Metabolism, Iran University of Medical Sciences (IUMS), Tehran, Iran; 10 Department of Internal Medicine, School of Medicine, Firoozgar Hospital, Iran University of Medical Sciences, Tehran, Iran; 11 Department of Pathology, School of Medicine, Center for Women’s Health Research Zahra, Tabriz University of Medical Sciences, Tabriz, Islamic Republic of Iran; 12 Research Center for Biochemistry and Nutrition in Metabolic Diseases, Kashan University of Medical Sciences, Kashan, Iran; 13 Student Research Committee, Kashan University of Medical Sciences, Kashan, Iran

**Keywords:** MicroRNAs, Let-7, Viral infections, Regulatory role

## Abstract

MicroRNAs (miRNAs) are fundamental post-transcriptional modulators of several critical cellular processes, a number of which are involved in host defense mechanisms. In particular, miRNA let-7 functions as an essential regulator of the function and differentiation of both innate and adaptive immune cells. Let-7 is involved in several human diseases, including cancer and viral infections. Several viral infections have found ways to dysregulate the expression of miRNAs. Extracellular vesicles (EV) are membrane-bound lipid structures released from many types of human cells that can transport proteins, lipids, mRNAs, and miRNAs, including let-7. After their release, EVs are taken up by the recipient cells and their contents released into the cytoplasm. Let-7-loaded EVs have been suggested to affect cellular pathways and biological targets in the recipient cells, and can modulate viral replication, the host antiviral response, and the action of cancer-related viruses. In the present review, we summarize the available knowledge concerning the expression of let-7 family members, functions, target genes, and mechanistic involvement in viral pathogenesis and host defense. This may provide insight into the development of new therapeutic strategies to manage viral infections.

## Background

MicroRNAs (miRNAs) are small (about 22 nucleotides) RNA molecules that have been shown to regulate gene expression in eukaryotic cells through various mechanisms [1–3]. First described in *Caenorhabditis elegans*, miRNAs have now been found to be widespread in nature. miRNAs have a central role in regulating a number of genes, particularly those genes involved in signaling pathways, and several physiological processes in human cells, including (but not limited to) cellular proliferation, lifespan, metabolism, and cell cycle control [4–6]. It is now believed that viruses, which exploit many elements of the host gene expression machinery, are able to encode miRNAs within their genome. Studies over the past decade have elucidated several important roles for viral miRNAs. Furthermore, several host-encoded miRNAs can potentially control viral replication by interacting with target sequences in viral RNAs [6, 7]. miRNAs could have a role as biomarkers of virus-related tumors, and also have some therapeutical potential in cancer treatment. Now, several miRNA-based treatments are being examined in preclinical and clinical trials, for instance, miR-122 in hepatitis C virus (HCV) infection [8]. Besides, virus-mediated changes in miRNA expression can provide an environment that facilitates tumor development [9]. Although some success has been achieved, further studies are still required to fully understand miRNA-based pathways, and virus-related miRNAs.

Extracellular vesicles (EVs) are a family of membrane structures that can be classified based on the vesicle size, function, RNA contents, or biogenesis. According to a classification by the International Society of Extracellular Vesicles, EVs are divided into subclasses including exosomes, microvesicles, and apoptotic bodies [10]. Due to the role of EVs as extracellular transporters of macromolecules, such as proteins and RNA transcripts, they have gained attention for a broad spectrum of applications [10]. EVs can package, release, and transfer miRNAs between cells in a somewhat selective manner. After uptake of the EVs by target cells, the miRNAs are actively released from the EVs. This process protects them against degradation by cell-free RNase enzymes. Viruses employ several mechanisms to evade and suppress the host immune responses, to ensure the establishment and maintenance of viral infections. Nevertheless, the host immune system uses opposing tactics to counteract the viral invasion [11]. Through evolution, viruses have developed the ability to incorporate their nucleic acid components into exosomes, which can exert downstream effects via various mechanisms [12]. Numerous experimental studies have reported the functional transfer of exosomal miRNAs between cells. This transfer facilitates virus replication through suppression of immune responses [6].

The lethal-7 (let-7) gene was first discovered in *C. elegans* as a key developmental regulator and was one of the first two known microRNAs. In mammals, let-7 is among the miRNAs with the highest expression level in numerous cell types in different species. Increasing evidence has shown the involvement of let-7 family members in critical physiological processes, such as organ development, growth, tissue regeneration, metabolism, and various types of cancer [13]. A vast number of miRNAs have been identified either as tumor inhibitors or as oncogenes (oncomiRs) according to the function of their target genes [4, 13]. Additionally, one individual miRNA can carry out paradoxical dual functions, by acting as a tumor inhibitor in one cancer type while acting as an oncomiR in another cancer type. Let-7 has been reported to possess broad tumor-suppressor effects in a range of cancer types [4]. Besides, many studies have reported the downregulation of let-7 in many viral diseases compared with healthy controls, and thus this miRNA may function as a putative factor encouraging the development of viral infections. Because of the many roles of let-7, modifications of its pathways could play a role in controlling viral infections [e.g., coronavirus disease 2019 (COVID-19), influenza, human immunodeficiency virus (HIV), etc.], as well as cancer-associated viruses. In the present review, we summarize the available knowledge about the expression of the let-7 family, its function, target genes, and mechanisms involved in viral infections, aiming to provide insight into its possible use in the control and therapy of viral infections.

## MicroRNA biogenesis and computational resources

**Fig. 1:**
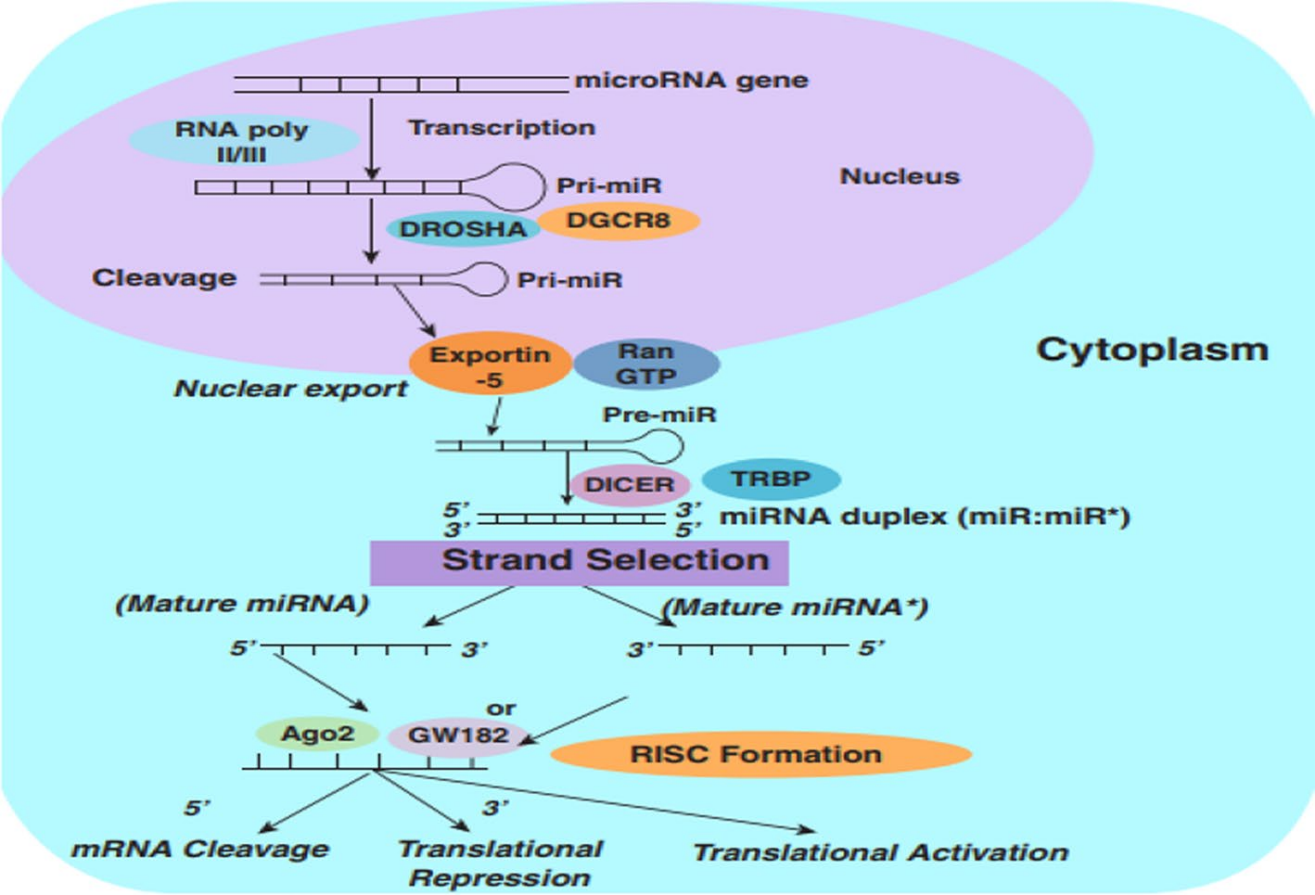
MicroRNA biogenesis. miRNAs are produced from miRNA genes, which are transcribed via RNA polymerase II/III to form primary miRNA termed pri-miRNA. Thereafter, pri-miRNA is cleaved by Drosha and DGCR8, and forms precursor microRNA (pre-miRNA), which is exported to the cytoplasm via Ran-GTP and exportin-5 to undergo maturation. miRNA duplex composed of mature miRNA is generated from cleaving the pre-miRNA, which is processed by Dicer and transactivation response element RNA-binding protein (TRBP). The single strand of mature miRNA, containing Ago-2 and GW182 proteins, binds to the complex, namely RISC. This complex modulates miRNA target gene expression by target miRNA cleavage and translation inhibition

Similar to other RNA transcripts, the biogenesis of miRNAs begins within the cell nucleus. The majority of miRNAs are initially generated as primary transcripts (pri-miRNAs) by RNA polymerase II, and then undergo further processing in the nucleus by RNase III Drosha, which forms long hairpin precursors about 70–100 nucleotides long (termed pre-miRNAs). Thereafter, the pre-miRNAs are exported to the cytoplasm to undergo further maturation. This involves the formation of a complex containing GTP-bound nuclear protein RAN GTPase, the pre-miRNA, and exportin 5 to allow the export of immature miRNAs [14]. After the pre-miRNAs are transported through the nuclear pore complex (NPC), the GTP is hydrolyzed, the NPC is disassembled, and the pre-miRNA is released into the cytosol [15, 16]. In the cytoplasm, the pre-miRNAs are cleaved by RNase III Dicer to form the mature miRNAs. This cleavage eventually leads to a double-stranded 22-nt product, composed of a mature miRNA guide strand and a passenger strand. The miRNA duplex is composed of two complementary strands called 5p and 3p. The passenger strand (annotated *) is typically degraded, whereas the opposite strand (guide strand) binds to the target mRNA sequence. The thermodynamic properties of the duplex seem to determine which strand is selected, because the strand with the weaker binding at the 5′ end of the duplex usually acts as the guide strand. Other essential properties of miRNA guide strands are a U bias at the 5′ end and a high percentage of purines (A/G rich), while the passenger strands possess a C bias at the 5′ end with a pyrimidine-rich (U/C) sequence. Nevertheless, the guide strand could tolerate a single point mutation within the duplex sequence, but this may affect post-transcriptional modification, and the type of proteins associated with Ago2 in the RNA-induced silencing complex (RISC) (e.g., trans-activation response RNA-binding protein versus protein activator of dsRNA-dependent protein kinase) [17–20]. Thus, both arms of the pre-miRNA hairpin can give rise to biologically functional guide miRNAs [21]. The miRNA guide strand then enters the RISC. Depending on the thermodynamic conditions, occasionally the passenger strand is also loaded into the RISC [22, 23]. The RISC binds to the target mRNA, due to the miRNA interacting with mRNA complementary sequences, resulting in target cleavage and/or translational inhibition (Fig. 1).

miRNAs are generally expressed at low levels, and this expression is different in various tissues and in certain environmental conditions. Considering their relatively small size, identifying miRNAs by their properties remains difficult, and experiments can be expensive, time consuming, and difficult. As a result, computational techniques have been proposed to effectively identify miRNA by their characteristics [24, 25]. Computational techniques take advantage of some features of miRNAs, including their conserved state among different species, the synthesis of stable stem-loop constructions from pre-miRNAs, and the minimum free energy of folding [26, 27]. Different computational approaches have been published in the previous decade. Computational resources containing expression profiles of miRNAs include human miRNA expression database (HMED) and mirEX 2.0 ([28, 29]. HMED and mirEX are considered to be comprehensive analysis programs for identifying datasets of miRNA expression. Moreover, some databases contain differentially expressed (DE) miRs obtained from many tissues; for instance, blood miRs are cell-specific miRNAs obtained from peripheral blood [30]. Likewise, ExCell miRDB is a specialized database containing curated information related to DEmiRs in biofluids [31]. Similarly, miRandola gives detailed information regarding several circulating extracellular miRNAs [32, 33]. In addition, many miRNAs have been identified as critical regulators of gene expression. Moreover, miRNA genes obtained from different species have been reported during the past decade, leading to the extensive expansion of miRBase, which is a core repository of miRNA sequences [34]. The current miRBase release version 21 contains a total of 2588 mature miRNAs and 1881 pre-miRNAs identified in the human genome (GRCh38). Moreover, miRNAs have also been identified in viruses. In a study by Tushcl et al., the miRNAs encoded by Epstein–Barr virus (EBV) were identified. Following this discovery, different viral miRNAs have been reported in other viruses [35, 36]. Currently, VIRmiRNA is a comprehensive database for experimentally verified viral miRNAs, containing 1308 different viral miRNA sequences [37]. A number of computational resources are focused on specific types of diseases; such as OncomiRDB, miRCancer, and dbDEMC, which are catalogs of deregulated miRNAs in different types of cancer [38, 39]. PhenomiR is an online database listing dysregulated miRNAs in different diseases and biological pathways [40]. Moreover, AVIRmiR is a subdatabase of VIRmiRNA [37], containing antiviral miRNAs that have been found to be involved in viral infections.

## MicroRNAs as biomarkers

The diagnostic potential of miRNAs has become the most important application in medicine. The term biomarker can involve different types of tests, which can provide information on normal or pathological processes [41]. Biomarkers are becoming more accurate and reliable as tremendous technological progress has recently been achieved. Historically, the first biomarkers were employed by early civilized humans, as simple signs such as pulse rate or urine appearance and odor. Currently, biomarkers often involve specific molecules, mainly proteins or small molecules, that can be found in different types of body fluids using analytical tools [42]. Nevertheless, developing novel protein biomarkers is a costly and time-consuming process that is limited by the lack of clinically significant proteins, structural complexity, and challenges in developing precise analytical methods. To achieve the goals of personalized medicine, novel biomarkers with higher accuracy are required. There are several requirements for developing a good biomarker. First and foremost, it should be readily accessible. In other words, a good biomarker should be detected and measured via minimally invasive procedures. Secondly, it should have the appropriate specificity and sensitivity to be utilized in clinical practice, meaning that the biomarker could be detected prior to the appearance of clinical symptoms, and also its levels would vary according to the clinical stage. Finally, an ideal biomarker should be translatable from the research to the clinical setting [43]. It has been found that free nucleic acids are present in human blood samples [44, 45], and DNA and RNA originating from tumor tissues are constantly released into the plasma of patients who are suffering from cancer [46, 47]. Previously, investigators believed that RNA molecules could not be employed as biomarkers by measurement in blood samples, due to the relatively high levels of nucleases in plasma that potentially degrade nucleic acids [48]. However, with the discovery of miRNAs that were stable in samples of fixed tissue, the idea was revisited [49]. For the first time, in 2008, Lawrie et al. reported that miRNAs could be used as cancer biomarkers. They demonstrated the diagnostic application of miRNAs in patients with diffuse large B-cell lymphoma [50, 51]. Ever since, many studies have been designed to assess the diagnostic ability of miRNAs in a wide variety of human disorders. miRNAs could be superior to conventional biomarkers in many human diseases. Moreover, miRNAs can be easily extracted from human bodily fluids, making them readily accessible. They also have shown satisfactory specificity for specific tissues or cell types and good sensitivity for disease progression state, and many studies have used miRNAs to differentiate different stages of cancer [52] and also for monitoring therapeutic responses [53]. Furthermore, there are already well-established techniques for the detection of DNA and RNA sequences, and the cost and time may be more economical, compared with developing new antibodies for detecting protein biomarkers. Additionally, miRNAs have shown potential for guiding the choice of different possible therapies, more precise diagnosis, and monitoring of the response to treatment. Multimarker panels that can measure several miRNAs at the same time could improve both the diagnosis and prognosis of cancers, as well as many other diseases [54, 55].

## Let-7

The lethal-7 (let-7) gene was initially described in *C. elegans* as a critical gene involved in embryonic development. Let-7 is a highly conserved miRNA across animal species, with 22 nucleotides in length. In *C. elegans*, the let-7 miRNA family includes nine members: let-7, mir48, mir-84, mir-241, mir-265, mir-793, mir-794, mir-795, and mir-1821, which serve to regulate developmental timing in a sequential order during the larval transition process [13, 56]. The main target genes of let-7 in *C. elegans* are *hbl-1*, *lin-41*, and *lin-42* [13]. Worms with mutations in let-7 show developmental timing abnormalities during the larval to adult transition [57, 58]. In *Drosophila melanogaster,* three miRNAs have been shown to be encoded by the let-7 complex (let-7C): let-7, fly lin-4 (miR-125), and miR100, which show the highest expression in the pupal and adult neuromuscular region. The function of let-7 in *Drosophila* is primarily to regulate the development transition from the third instar to the pupal stage. Flies with mutations in let-7C showed abnormalities in adult behavior (flight, motility, and fertility), and in juvenile neuromusculature features; however, they appeared normal on external examination. Let-7C has been shown to cause programmed remodeling of the abdominal neuromusculature during larval to adult transition [59]. In mice and humans, the let-7 family has 14 and 13 members, respectively. In humans, these members are let-7a-1, let-7a-2, let-7a3, let-7b, let-7c, let-7d, let-7e, let-7f-1, let-7f-2, let-7g, let-7i, mir-98, and 202. Among these, let-7a has the most conserved sequence across different animal species from nematode *C. elegans* to *Homo sapiens*. In mammals, the expression levels of let-7 are highest during embryogenesis and central nervous system (CNS) development. Nevertheless the let-7 family is not seen in human or mouse embryonic stem cells [13, 60].

## Let-7 functions

In most organisms, let-7 expression is increased in the late stages of development, and any alteration in the expression of let-7 can lead to pathological conditions, such as neurodegenerative diseases, cancer, and diabetes [60]. Let-7 is also involved in the regulation of cell signaling pathways. Overexpression of miR-98 inhibited the phosphorylation and repressed the Akt and ERK signaling pathways, which are well known to be implicated in carcinogenesis [61–63]. In Ewing’s sarcoma, it was found that let-7 directly downregulated signal transducer and activator of transcription 3 (STAT3), consequently reducing the aggressive phenotype [64]. The STAT3 pathway controls cell cycle and cell survival via regulation of a specific gene set, and so any overactivation can drive cancer progression. Poor outcomes and enhanced resistance to chemotherapy and radiotherapy have been observed following activation of the STAT3 signaling pathway [65, 66]. Let-7 has been reported to activate the WNT signaling pathway via targeting estrogen receptors in breast cancer, and transcription factor 4 (TCF-4; a downstream target of WNT) in hepatocellular carcinoma (HCC), resulting in increased cancer stemness and aggressiveness [67]. The WNT pathway plays a central role in several cellular process, including differentiation, proliferation, and migration, and has been shown to enhance tumor growth and the cancer stem cell phenotype [68, 69]. The data suggests that let-7 prevents the aggressive phenotype via negative regulation of carcinogenic signaling pathways [65].

An anti-let-7 2′-*O*-Me oligonucleotide increased cancer cell proliferation, highlighting the fact that, in human cells, let-7 exerts its tumor suppressor effects via inhibition of pathways involved in cell proliferation [70]. In another experiment, Johnson et al. demonstrated that let-7 plays a fundamental role in malignancy. The authors reported that let-7 family members acted as tumor suppressors via negative regulation of the let-60/RAS axis [71]. Let-7 inhibited the expression of a set of oncogenes and other genes involved in tumor development and progression. These included RAS, LIN28, PBX3, E2F5, E2F1, Myc, ARID3B, long noncoding RNA (lncRNA) H19, and HMGA2 [61, 63, 72, 73]. Silencing experiments using specific antisense oligonucleotides (ASOs) showed that downregulation of these genes led to tumor suppression, which would normally depend on let-7 [65]. Lan et al. suggested that let-7 acted as a tumor suppressor [74]. They found inhibition of proliferation in HepG2 HCC cells overexpressing let-7g, by repression of the oncogene c-Myc. Gene expression analysis showed a decrease in the corresponding mRNAs and protein levels. Transfection of the cancer cells with a let-7g inhibitor reversed the effects of miRNA overexpression [74]. Let-7g upregulation also enhanced expression of the p16INK4A tumor suppressor protein, indicating that the let-7g tumor suppressor effect is probably due to miRNA direct control of c-Myc in the regulatory axis (c-Myc-Bmi-1-p16) [75, 76]. These data suggest that let-7g may play its role as a tumor inhibitor in HCC through directly repressing the c-Myc oncogene, leading to increased expression of p16INK4A with tumor suppressor effects [74]. Therefore, let-7 is able to suppress the function of several factors involved in oncogenesis.

Paradoxically, let-7 also has shown some pro-oncogenic properties. Although most of the evidence supports the fact that let-7 is a tumor inhibitor in several cancer types, some new studies have suggested let-7 can function as an oncogene. Several studies have shown that the let-7a3 locus is highly methylated in healthy tissue compared with its hypomethylation status in cancer tissues such as lung and ovarian cancer. Moreover, increased expression of mature let-7a has been observed in these cancers [65, 77, 78]. Brueckner et al. showed that let-7a3 overexpression in lung cancer cells caused more aggressive behavior in an anchorage-independent culture experiment. They found alteration in the expression of several factors controlling cell proliferation, and also a number of genes associated with cell adhesion, encouraging tumor progression and metastasis [77]. Also, higher values of several let-7 family members, such as let-7a3, let-7c, and let-7b, were found to be significantly associated with poor prognosis and short overall survival in patients with ovarian and liver cancer [65]. Ma et al. reported that the increased expression of let-7e in esophageal squamous cell carcinoma (ESCC) cells, increased migration and invasion probably through downregulation of the downstream transcription factor ARID3a. ARID3a acts as a negative regulator of pluripotency, so its downregulation may lead to cancer stemness [79, 80]. The let-7 family member mir-98 has been shown to increase chemoresistance in cancer cells via negative regulation of mir-152 mediated by repression of Dicer1. mir-152 and high expression of miR-98 can regulate RAD51 recombinase levels, which were correlated with poor prognosis in patients with epithelial ovarian cancer (EOC) [65, 81]. Overall, these findings highlight the complex relationship between let-7 and cancer cell aggressiveness, underlining the context-dependent role of many miRNAs. It is possible that let-7 in each specific cancer cell targets a set of genes that are particularly expressed in that cell type. Therefore, using an individual miRNA expression profile to develop a “personalized medicine” approach to target let-7 expression in each patient could be necessary.

When any type of infectious agent enters the body, the innate immune system is the first to be activated, which is able to differentiate microbial cells from the host cells [82]. Toll-like receptors (TLRs) play substantial roles in the recognition of invading pathogens and trigger inflammatory responses designed to prime specific adaptive responses to each type of infection. Dysregulation of TLR signaling pathways can promote excessive inflammation, and contribute to the development of diseases, including various types of cancer [83]. Many miRNAs have been identified as key regulators of TLR signaling [84, 85]. Let-7 seems to play a substantial role in the regulation of TLR4 signaling. For instance, after *Cryptosporidium parvum* infection, let-7i expression was lower, while TLR4 signaling was upregulated [86]. This finding indicates that let-7i may contribute to host immune responses by modulating the *C. parvum*-induced upregulation of TLR4. In another study, the induction of TLR4 signaling in response to *Helicobacter pylori* infection was linked with let-7b. TLR4 controls the activation of nuclear factor κB (NF-κB) and the expression of a set of downstream genes involved in inflammation. Let-7 directly targets the TLR4 mRNA to repress translation, and reduces the innate immune response and inflammation after infection [87].

Let-7 also contributes to the function of adaptive immune cells. Let-7 expression affects the differentiation of effector CD8 T cells, which can release effector cytokines and eliminate the infected target cells. Moreover, let-7 affects the differentiation of single positive thymocytes into naive or memory-like CD8^+^ cytotoxic T lymphocytes (CTLs). In activated CTLs, lowered let-7 levels enhance the clonal expansion and the acquisition of effector functions via negative regulation of its target genes, Myc and eomesodermin (Eomes) [88]. Let-7 can also regulate the promyelocytic leukemia zinc finger (PLZF) transcription factor, which affects the differentiation and effector functions in natural killer T (NKT) cells [89, 90]. Hence, let-7, by repressing PLZF, can control the cellular development of the thymus, activation of B cells, and antibody production [89].

## Let-7 and human oncoviruses

Among the approximately 1400 known human pathogens, viruses make up the largest group. Several viruses are carcinogenic, and can induce various cancers in infected patients [91]. Viruses have been implicated in the causation of approximately 14% of cancers, including human papillomavirus (HPV), hepatitis C virus (HCV), hepatitis B virus (HBV), and human herpes virus 8 (HHV8). HBV or HCV are responsible for 80% of HCC cases, the most frequent primary liver malignancy. Some types of HPV, known as high-risk types, are the underlying cause of cervical cancer, as well as several types of head and neck carcinoma. HHV8 and Kaposi’s sarcoma-associated herpesvirus (KSHV) have been identified as causative agents for Kaposi’s sarcoma, which is typically found in HIV-infected patients. HHV8 is also implicated in the pathogenesis of two uncommon B-cell cancers. It has been estimated that HPV is the leading cause of virus-associated cancer worldwide, accounting for about 600,000 new cases every year. On the other hand, HTLV is the least common cause of virus-associated cancer, with 2100 new cases every year. The majority of virus-associated cancers are seen in developing countries, which highlights the crucial need for public health intervention in those regions [92].

### Hepatitis B and C viruses

Hepatocellular carcinoma (HCC) is the most common (85%) among primary liver cancers. Globally, HCC is the sixth most common malignancy and the second highest cause of cancer-related death [93]. Chronic HBV infection is the main underlying cause of HCC in Asian countries, while other causes including chronic HCV infection, alcoholic cirrhosis, and non-alcoholic steatohepatitis (NASH) are the major risk factors in Western countries. Other risk factors are excess alcohol consumption and nonalcoholic fatty liver disease [93, 94]. HBV causes cancer by several mechanisms, including virus DNA integration into chromosomes, epigenetic alterations like methylation, oxidative stress, and HBV transcriptional activator HBx protein [95].

HBV DNA and hepatitis B e antigen (HBeAg) can be measured in the blood circulation of HBV-infected patients, and are currently employed in laboratory analysis of affected patients. However, neither can be used as a surrogate marker of viral infection progression and carcinogenesis in clinical settings, because of the heterogeneous nature of HCC. Unfortunately, measurement of the viral load and clinical manifestations are not able to predict the clearance rate and the prognosis in patients with persistent infection. Hence, to optimize the management of HVB-associated diseases, it is important to identify biomarkers and host genetic risk factors, as well as viral and environmental factors. Besides, the occurrence of HCC in patients with cirrhosis is difficult to diagnose, due to the lack of early symptoms [96, 97].

The importance of miRNAs in HCC has been shown by the measurement of miRNA profiles with differential expression in HCC cell lines and tissues compared with normal counterparts [98–101]. These pioneering studies provided a rationale for examining molecular mechanisms, developing improved diagnostic procedures, and exploring novel therapeutic targets in HCC. High quantities of stable miRNAs have been detected in the circulation in a number of studies, suggesting that differentially expressed miRNAs may serve as reliable fingerprints for many human diseases [4, 5, 102, 103]. For instance, Li et al. [104] examined the hypothesis that the expression profiles of miRNAs could serve as a surrogate marker for the diagnosis of HBV infection, and HBV-positive HCC cases. In this study, 513 patients with HBV (*n* = 135), HCV (*n* = 48), and HCC (*n* = 120) underwent primary screening by the Solexa sequencing procedure, validated by TaqMan probe-based quantitative reverse-transcription PCR (qRT-PCR). The results showed upregulation of both miR-25 and let-7f in plasma samples from patients with HCC; however, no changes were seen in HBV-infected serum samples, and occasionally decreased expression was seen. The authors suggested that some upregulated miRNAs may be involved in HCC development, independent of chronic HBV infection, and could be valuable biomarkers for both HBV infection and HBV-positive HCC patients. Additionally, they reported that miR-375, miR-25, and let-7f showed acceptable receiver operating characteristic (ROC) curves [area under the curve (AUC) of 99.67, sensitivity of 97.9%, and specificity of 99.1%] to distinguish HBV-positive HCC patients from healthy individuals [104].

It was initially believed that HBV could only be transmitted in blood that was positive for the hepatitis B surface antigen (HBsAg); however, it was later shown that the virus could be transmitted by HBsAg-negative blood samples during the seronegative window of the acute infection phase, or in the chronic phase, in the case of occult hepatitis virus B infection (OBI) [105]. HBV DNA can remain in the blood or liver tissue of patients with OBI who were diagnosed as negative for HBsAg. Some patients with OBI may test positive for anti-HBV core antigen- (anti-HBc); therefore, anti-HBc has been employed in screening tests for OBI in blood donors. Nevertheless, it has been shown that > 20% of those who are negative for all virus markers still carry occult HBV infection [106]. Since the main strategy for detection of HBV uses HBsAg, the occult carriers might escape routine screening tests and go on to transmit the virus. Accurate detection of OBI cases is crucial for the elimination of potential viral transmission via blood transfusions [107, 108]. Because there is a possibility that OBI patients are negative for HBsAg and other biomarkers, there is a need for an accurate test for detection of the virus that is equivalent to the HBV DNA PCR assay [109]. Chen et al. [110] used qRT-PCR assays to compare the expression of 13 different HBV-associated miRNAs in serum samples obtained from 11 patients with OBI and 29 healthy subjects. The authors showed that patients with OBI had significantly higher values of miR-23b, miR-150, let-7c, and miR-122 relative to normal subjects. On ROC curve analysis, a signature profile of these four miRNAs could discriminate healthy individuals from OBI patients with an AUC value of 99.9, sensitivity 99.9%, and specificity 99.8% [110] (Table 1).

**Table 1:** Role of let-7 family members as biomarkers in viral infections

Virus	Let-7 member	Expression	Method	Sample	ROC (sensitivity/specificity %)	Refs.
HBV	Let-7c	Up	qRT-PCR	Human (serum samples of chronic hepatitis, *n* = 29)	99.1/98.8	[110]
HBV	Let-7c	Up	qRT-PCR	Human (serum samples of OBI, *n* = 11)	99.9/99.8	[110]
HBV	Let-7f	Up	Solexa Sequencing qRT-PCR	Human (serum samples of HCC, *n* = 55)	97.9/99.1	[104]
HBV	Let-7b	Up	qRT-PCR	Human (serum samples of early HCC, *n* = 120)	84.8/50	[273]
HBV	Let-7d-5p	Up	qRT-PCR	Human (serum samples of fibrosis, *n* = 14)	AUC = 0.82	[274]
HBV	Let-7c	Up	Solexa Sequencing qRT-PCR	Human (serum samples of chronic HBV, *n* = 30)	–	[104]
HBV	Let-7c	Up	qRT-PCR	Human (serum samples of chronic HBV, *n* = 30)	–	[104]
HBV	Let-7b-3p	Up	Microarray	Human (PBMC samples of chronic HBV, *n* = 16)	–	[275]
HBV	Let-7a	Up	qRT-PCR	Human (tissue samples of hepatocellular carcinoma with active virus replication, *n* = 13) /in vitro (HepG2.2.15)	–	[114]
HBV	Let-7a, b, c	Up	qRT-PCR	Human (tissue samples of chronic HBV)	–	[276]
HBV	Let-7g	Up	qRT-PCR	Human (tissue samples post-treatment with nucleos(t)ide analog in chronic HBV)	–	[277]
HBV	Let-7g	Up	qRT-PCR	Human (*n* = 14 tissue samples of hepatocellular carcinoma with post-treatment nucleos(t)ide analog)	–	[277]
HBV	Let-7a, b, d, g, i	Up	Microarray	In vitro (chronic hepatitis HepG2.2.15)	–	[278]
HBV	Let-7b	Down	qRT-PCR	Human (serum samples of chronic HBV with dysplastic nodule, *n* = 30)	84.8/50	[273]
HBV	Let-7f	Down	qRT-PCR	Human (serum samples of HCC, *n* = 373)	–	[279]
HBV	Let-7c Let-7a	Down	qRT-PCR	Human (tissue samples of HCC, *n* = 23)	–	[280]
HBV	Let-7a	Down	qRT-PCR	Human (tissue samples of HCC, *n* = 20)	–	[124]
HBV	Let-7c	Down	qRT-PCR	Human (tissue samples of HCC, *n* = 25)	–	[281]
HBV	Let-7a	Down	qRT-PCR	Human (tissue samples of HCC, *n* = 20)	–	[282]
HBV	Let-7a, b, c, d	Down	Sequencing qRT-PCR	Human (tissue samples of HCC)	–	[283]
HBV	Let-7a	Down	qRT-PCR	Human (tissue samples of HCC with less active virus replication, *n* = 10) /in vitro (HepG2)	–	[114]
HBV	Let-7g	Down	qRT-PCR	Human (tissue samples of HCC with pretreatment nucleos(t)ide analog, *n* = 15)	–	[277]
HBV	Let-7g	Down	qRT-PCR	Human (tissue samples of pretreatment with nucleos(t)ide analog in chronic HBV, * n* = 27)	–	[277]
HBV	Let-7	Down	qRT-PCR	Human (tissue samples of HCC, * n* = 19) /in vitro (HBx-HepG2)	-	[284]
HBV	Let-7a, c, d, e, f, g, i	Down	Microarray qRT-PCR	In vitro (HBx-HepG2)	–	[124]
HBV	Let-7a, b, c, e, i	Down	Microarray qRT-PCR	In vitro (HBx-SNU-182)	–	[124]
HBV	Let-7a, g	Down	Microarray	In vitro (acute hepatitis HepG2)	–	[278]
HBV	Let-7a	Down	qRT-PCR	In vitro (HepG2)	–	[120]
HBV	Let-7f	-	Microarray qRT-PCR	Human (plasma samples of chronic HBV treated with PEG-IFN, *n* = 94)	–	[285]
HCV	Let-7a-1	Up	qRT-PCR	Human (serum samples of chronic HBV with liver cirrhosis, *n* = 20)	75/70	[134]
HCV	Let-7c, g, i	Up	qRT-PCR	Human (serum samples of HCV, *n* = 33)	–	[286]
HCV (genotype 1)	Let-7g	Up	qRT-PCR	Human (tissue, serum samples of chronic HCV treated with PEG-IFN/RBV, *n* = 18)	–	[287]
HCV	Let-7a-5p	Down	qRT-PCR	Human (serum samples of chronic HCV with liver cirrhosis, *n* = 25)	92/80	[133]
HCV	Let-7a-1	Down	qRT-PCR	Human (serum samples of HCC, *n* = 40)	70/82.5	[134]
HCV	Let-7d-5p	Down	qRT-PCR	Human (plasma samples of chronic HCV with liver fibrosis, *n* = 24)	AUC = 0.79	[262]
HCV	Let-7a-5p	Down	qRT-PCR	Human (plasma samples of chronic HCV with liver fibrosis, *n* = 24)	AUC = 0.77	[262]
HCV	Let-7c-5p	Down	qRT-PCR	Human (plasma samples of chronic HCV with liver fibrosis, *n* = 24)	AUC = 0.73	[262]
HCV	Let-7a-5p Let-7c-5p Let-7d-5p	Down	Microarray qRT-PCR	Human (plasma samples of chronic HCV, *n* = 32)	–	[262]
HCV	Let-7a, b, c, d, e, g	Down	qRT-PCR	Human (plasma samples of chronic hepatitis, *n* = 236)	–	[288]
HCV	Let-7a	Down	qRT-PCR	Human (serum samples of HCV, *n* = 97)	–	[289]
HCV	Let-7a-1	Down	qRT-PCR	Human (serum samples of chronic HCV, *n* = 20)	-	[134]
HCV (genotype 1)	Let-7g	Down	qRT-PCR	Human [tissue (*n* = 6), serum (*n* = 19) samples of chronic HCV untreated with PEG-IFN/RBV]	–	[287]
HCV (genotype 1)	Let-7g	Down	qRT-PCR	Human (tissue samples of chronic HCV, *n* = 18)	–	[287]
HCV (genotype 4)	Let-7a, g	Down	qRT-PCR	Human (tissue samples of chronic HCV, *n* = 50)	–	[290]
HCV	Let-7i	Down	qRT-PCR	Human (tissue samples of HCC, *n* = 22)	–	[291]
HCV	Let-7a, b, c, d, e, f, g, i	Down	qRT-PCR	Human (tissue samples of transplanted liver with HCV-related end-stage liver disease, *n* = 61)	–	[140]
HCV	Let-7a, b, c, d	Down	Sequencing qRT-PCR	Human (tissue samples of HCC)	–	[283]
HCV (genotypes 1b and 2a)	Let-7g	Down	qRT-PCR	In vitro (Ava.5-Huh7, JFH1-Huh7.5.1)	–	[287]
HTLV-I	Let-7a	Up	qRT-PCR	In vitro (Tax-Hut102)	–	[292]
HTLV-I	Let-7a	Down	qRT-PCR	Human (blood samples of adult T-cell leukemia) /in vitro (HBZ-C81-66, ATL-2)	–	[292]
HHV-8	Let-7a, b, c, d, e, f, g, i	Down	Microarray	Human (tissue samples of Kaposi’s sarcoma, *n* = 14)	–	[293]
HHV-8	Let-7a, b, e, f	Down	qRT-PCR	Human (tissue samples of Kaposi’s sarcoma, *n* = 4)	–	[294]
HHV-8	Let-7a, b, e, f	Down	qRT-PCR	Human (tissue samples of primary effusion lymphoma, *n* = 12)	–	[294]
HHV-8	Let-7a	Down	qRT-PCR	In vitro (TIVE cell)	–	[295]
HPV-16	Let-7a	Up	qRT-PCR	Human (tissue samples of precervical cancer (LSIL), *n* = 4)	–	[192]
HPV-16 and -18	Let-7a	Up	qRT-PCR	Human (tissue samples of precervical cancer (HSIL), *n* = 9)	–	[192]
HPV-16	Let-7d	Down	Microarray qRT-PCR	Human (tissue samples of head and neck squamous cell carcinoma, *n* = 37)	–	[296]
HPV	Let-7b	Down	qRT-PCR	Human (serum, brush pap samples of squamous cervical cell carcinoma, *n* = 7)	–	[297]
HPV	Let-7g	Down	qRT-PCR	Human (tissue samples of cervical cancer, *n* = 20)	–	[298]
HPV	Let-7	Down	qRT-PCR	Human (tissue samples of lung cancer, *n* = 56)	–	[103]
EBV	Let-7d-5p Let-7f-5p	Up	Illumina deep sequence	In vitro (SNK6 cell)	–	[299]
EBV/HSV-2	Let-7b	Up	qRT-PCR	Human (serum samples of sex workers, *n* = 46)	–	[300]
EBV/ HPV	Let-7b	Up	qRT-PCR	Human (serum samples of sex workers, *n* = 5)	–	[300]
EBV	Let-7a-5p Let-7c-5p Let-7d-5p Let-7e-5p Let-7g-5p	Down	qRT-PCR	Human (plasma samples of mononucleosis, *n* = 15)	–	[301]
EBV	Let-7c Let-7e	Down	qRT-PCR	Human (tissue samples of post-transplant smooth muscle tumor, *n* = 5)	–	[302]
EBV	Let-7a-5p Let-7g-5p	Down	Illumina deep sequence	In vitro (SNK6 cell)	–	[299]
EBV	Let-7a-5p Let-7g-5p Let-7i-5p	Down	Illumina deep sequence	In vitro (SNT16 cell)	–	[299]
EBV	Let-7a-5p :et-7b-5p Let-7f-5p	Down	Sequencing qRT-PCR	In vitro (AGS)	–	[303]
EBV	Let-7b	Down	qRT-PCR	Human (serum samples of sex workers, *n* = 15)	–	[300]
HHV-6A	Let-7c	Down	Microarray	In vitro (NK-92 cell)	–	[304]
HHV-6B	let-7c	Down	Microarray	In vitro (NK-92 cell)	–	[304]
HSV-2	Let-7b	Up	qRT-PCR	Human (serum samples of sex workers, *n* = 67)	–	[300]
SARS-CoV-2	Let-7b	Up	qRT-PCR	Human (PBMC samples of COVID-19, *n* = 18)	83.3/93.3	[305]
RSV	Let-7d	Up	Microarray qRT-PCR	Human (nasal mucosa samples, *n* = 42)	–	[306]
RSV	Let-7b	Up	qRT-PCR	In vitro (MDDC cell)	–	[307]
RSV	Let-7c, i	Up	qRT-PCR	In vitro (NHBE cell)	–	[307]
H1N1	Let-7e Let-7f	Up	Microarray	In vivo (mice)	–	[207]
H1N1	Let-7a, e, f, g, i	Up	NGS	In vitro (A549 cell)	–	[308]
H7N9 Avian	Let-7b, g	Up	qRT-PCR	Human (serum sample, *n* = 21)	–	[309]
H5N1	Let-7a, b, e, f	Up	NGS	In vitro (A549 cell)	–	[308]
H3N2	Let-7b, g, f	Up	NGS	In vitro (A549 cell)	-	[308]
H1N1	Let-7g	Down	Microarray qRT-PCR	Human (PBMC samples, *n* = 299)	–	[208]
H1N1	Let-7g	Down	qRT-PCR	In vitro (A549)	–	[310]
H7N9	Let-7e	Down	qRT-PCR	Human (serum samples, *n* = 21)	–	[309]
H7N7	Let-7g	Down	qRT-PCR	In vitro (A549 cell)	–	[310]
H5N1	Let-7g	Down	NGS	In vitro (A549 cell)	–	[308]
H5N1 Avian	Let-7f	Down	Microarray	In vivo (macaque)	–	[311]
H3N2	Let-7a, i	Down	NGS	In vitro (A549 cell)	–	[308]
HIV-1	Let-7g-3p	Up	qRT-PCR	Human (plasma samples of acute HIV-1, *n* = 60)	100/100	[240]
HIV-1	Let-7g-3p	Up	qRT-PCR	Human (plasma samples of eclipse HIV-1, *n* = 20)	100/95.8	[240]
HIV-1	Let-7b, I, f	Down	Microarray qRT-PCR	Human (PBMC samples of chronic HIV, *n* = 7)	–	[123]
HIV-1	Let-7c	Down	qRT-PCR	Human (plasma samples of naive HIV-1, *n* = 25)	–	[312]
HIV-1	Let-7c	Down	qRT-PCR	Human (plasma samples of HIV with ART therapy, *n* = 25)	–	[312]
HIV-1	Let-7c	Down	qRT-PCR	Human (plasma samples of elite controller, *n* = 19)	–	[312]
HIV-1	Let-7g	Down	Nanostring TLDA	Human (PBMC samples of untreated-viremic controller, *n* = 6)	–	[313]
Human Metapneo	Let-7f	Up	qRT-PCR	In vitro (A549 cell)	–	[314]
West Nile	Let-7a, e, g, i	Up	qRT-PCR	In vivo (mice)	–	[315]
West Nile	Let-7c	Down	qRT-PCR	In vivo (mice)	–	[315]
Japanese encephalitis	Let-7a, b	Up	qRT-PCR	Human [*n* = 3 tissue samples of encephalitis/in vitro (N9)/in vivo (mice)]	–	[270]
DENV-2	Let-7e	Down	qRT-PCR	In vitro (PBMC)	–	[316]
Zika	Let-7a	Down	NGS	In vitro (neural stem cell)	–	[317]
Hendra	Let-7	Up	NGS	Horse (*n* = 6 blood samples)	–	[318]
Persistent Coxsackie B4	Let-7b-3p Let-7d-3p Let-7f-1-3p	Down	Sequencing	In vitro (PANC-1)	–	[319]

It has been repeatedly shown that let-7 miRNAs are associated with viral infection, dysfunction of liver cells, and immune response. For example, Shimizu et al. [111] observed that overexpression of let-7 miRNA family members (let-7c or let-7g) led to a significant decrease in anti-apoptotic protein Bcl-xL in two HCC cell lines. This finding suggested that let-7 induced apoptosis via repression of Bcl-xL expression in human HCC. Additionally, OBI can lead to the development of HCC by three possible mechanisms, including production of pro-oncogenic proteins, chronic inflammation leading to hepatic necrosis, and the integration of virus DNA into human liver DNA [112]. Thus, overexpression of let-7 could play a role in the pathogenesis of OBI-related HCC via increasing liver cell damage.

Furthermore, some studies have reported lower values of let-7a in tissues of patients with HCC who are positive for HVB infection compared with healthy controls (Table 1). It has been proposed that let-7a can suppress hepatocyte proliferation by affecting the USP35-ABIN-2 signaling pathway [113], thereby acting as a tumor suppressor in HCC. Recently, Qiu et al. [114] showed that HCC tissue samples expressed let-7a in significantly lower values compared with adjacent normal liver tissue. Moreover, they found higher tissue values of let-7a in patients with highly active HBV replication (HBV DNA > 10^6^ copies/mL) relative to those with less active HBV replication (HBV DNA < 10^3^ copies/mL). In addition, knockdown of let-7a in HepG2.2.15 cells (HepG2 cells engineered to overexpress let-7) using an antisense oligonucleotide resulted in a significant decrease in HBV DNA copy numbers, demonstrating a positive correlation between let-7a and HVB replication. The authors suggested that high levels of let-7a could suppress HCC invasion and proliferation, while it was paradoxically able to enhance HBV replication in hepatocytes [114]. On the other hand, Takata and colleagues [115] reported that mRNA coding for the HBsAg preS2 region in HBV was targeted by the host miRNA let-7g. The expression of HBV mRNAs, such as the preS2 region, could lead to de-repression of the let-7g targets, which could result in long-term oncogenesis. Conversely, let-7g was shown to inhibit the expression of the HBV preS2 protein and other viral proteins. These findings suggested that interactions between the HBV transcripts and the host miRNAs may play a role in the pathogenesis of chronic viral hepatitis [115]. Further studies have reported a negative correlation between the intrahepatic pre-S2 mRNA expression levels and let-7a values [116]. Deng et al. observed that HBV mRNAs repressed let-7a via the miRNA response element, which could lead to universal de-repression of the let-7a host mRNA targets. These changes may contribute to transformation of HCC cells and tumor growth [116]. It is expected that the identification of reciprocal relationships between the viral and host mRNAs may provide insight into HCC pathogenesis, and could inform new therapies against this malignancy.

To date, several studies have shown that the HBV HBx protein can modulate miRNA expression via three main pathways. Firstly, HBx can interact with a transcription factor that regulates miRNA expression [117–119]. Secondly, the HBx mRNA can act as a sponge for host miRNAs [120, 121]. Lastly, HBx has been suggested to modulate the biogenesis of pri-miRNA by decreasing the protein levels of Drosha RNase. However, other studies have shown upregulation of several regulators of miRNA biogenesis, such as Drosha, DGCR8, Ago1, and Ago2 [122, 123]. Wang et al. evaluated the expression levels of miRNAs in HBx-expressing cells relative to HepG2 cells (control), using miRNA microarrays. They observed that HBx led to overexpression of 7 miRNAs, while it downregulated 11 other miRNAs, including the let-7 family [124]. HBx-mediated let-7a repression was shown to enhance tumor cell proliferation and promote hepatocarcinogenesis by increasing the expression of transcription factor STAT3. Moreover, it was demonstrated that HBx also downregulated let-7i, which in turn controlled the expression of complement system regulator CD59. Through this mechanism, HBx could protect the HCC cells against complement-dependent cytotoxicity [125]. Overall, HBV proteins have been suggested to contribute to HCC pathogenesis via negative regulation of the let-7 miRNA family members.

HCV accounts for 140,000 new cases of HCC annually [126]. Due to delayed diagnosis, most patients have a poor prognosis; thus, an early diagnosis could improve the survival of many HCC patients [127–130]. Current HCC guidelines recommend imaging-based diagnosis as the only standard procedure [131, 132]. In this context, much effort has been devoted to find predictive noninvasive biomarkers for the early diagnosis of HCC, and miRNAs are good candidates for this role. Thus, the discovery of predictive biomarkers for the diagnosis and monitoring of HCC is regarded as an urgent issue. Matsuura et al. [133] assessed whether there was any correlation between values of circulating let-7a-5p and the severity of hepatic fibrosis in chronic hepatitis C (CHC) patients. They measured circulating let-7a-5p in serum samples, and in serum-derived extracellular vesicles (EVs) retrieved by a liver biopsy in 84 Japanese patients diagnosed with CHC by qRT-PCR. They investigated the possible correlation between let-7a-5p values and clinicopathological features (histological fibrosis grade, markers of hepatic fibrosis, liver stiffness) in the recruited patients. They found that the circulating levels of let-7a-5p were remarkably lower in patients who were diagnosed with liver cirrhosis. More importantly, transient elastography showed that let-7a-5p serum levels were significantly associated with liver stiffness and hepatic fibrosis markers, including platelet ratio index (APRI), Mac-2 binding protein glycan isomer (M2BPGi), and fibrosis 4 (FIB-4). The ROC curve analysis demonstrated that serum let-7a-5p levels were better for diagnosis of cirrhosis compared with any of the other markers (AUC values of 0.892, 0.800, 0.788, and 0.783 for let-7a-5p, M2BPGi, APRI, and FIB-4, respectively) and were similar to measurement of liver stiffness (AUC 0.909). However, let-7a-5p levels in EVs (AUC 0.681) were lower compared with those in serum. As a result, the authors suggested that circulating let-7a-5p could be a biomarker for predicting the severity of hepatic fibrosis in patients suffering from CHC [133]. Aly et al. measured the expression pattern of the let-7 cluster, including let-7d-1, let7-a-1, and let-7f-1, in serum samples of patients with HCC or chronic HCV infection [134]. The authors found that the serum let-7a-1 levels were remarkably lower in the patients with HCV–HCC compared with the HCV cirrhotic group without HCC. It was also significantly increased in patients with liver cirrhosis compared with the HCV non-cirrhotic group. Additionally, ROC analysis showed that serum let-7a-1 could be a superior biomarker for liver cirrhosis development compared with HCV detection (let-7a-1 AUC 0.768; *p* = 0.004). They hypothesized that the lower expression of let-7-a1 in serum could promote the development of HCC in chronic HCV patients [134]. However, more studies are needed to assess the clinical application of let-7 detection to diagnose hepatic fibrosis in clinical settings.

miRNA let-7b has been shown to markedly suppress HCV viral replication, and has demonstrated a synergistic effect in combination with the antiviral cytokine interferon-α-2a (IFN-α-2a) in inhibition of HCV infection [135]. Bioinformatics analysis has revealed binding sites for let-7b on the coding region of NS5B and the 5′-untranslated region (UTR) of the HCV genome, which were conserved among different genotypes. Several studies have shown that let-7b is negatively correlated with viral replication and accumulation of HCV RNA, which was not dependent on inhibition of HCV translation. As far as we know, let-7b is the first known miRNA to contain a target site within the coding sequence of the HCV genome.

It was found that let-7b could modulate the expression of IFN-α and IL-28B, and also exert an antiviral effect through repression of HCV protein translation and replication. This was dependent on the host factor, insulin-like growth factor 2 mRNA-binding protein 1 (IGF2BP1). They also showed that there was a correlation between repression of let-7b and abrogation of the anti-viral effects of IL-28B and IFN-α. Moreover, IL-28B and IFN-α were shown to downregulate IGF2BP1 expression (a target of let-7b), leading to increased antiviral activity of let-7b [136, 137]. Recently, Chen et al. [138] reported that miR-let-7c overexpression significantly repressed the replication of HCV by stimulation of heme oxygenase-1 (HO-1) expression because it inhibited the transcriptional repressor Bach1, eventually resulting in an increased interferon response and suppression of viral protease activity. Accordingly, treatment with a specific inhibitor, exogenous expression of Bach1, and suppression of HO-1 activity and expression all reduced the antiviral activity of miR-let-7c. Taken together, these results highlight the key role of let-7c against HCV infection via targeting Bach1 and consequent transactivation of HO-1-mediated antiviral activity, suggesting a possible role of of let-7c as an antiviral treatment [138].

It has been proposed that let-7a and let-7b can inhibit HCV infection by modulating several cofactors necessary for HCV cell entry, protein production, and RNA replication [139]. There was a negative correlation between the let-7/miR-98 expression levels and the HCV viral load in liver transplantation patients [140]. Upon infection with HCV, some host immune response factors could be modulated by let-7. As previously stated, the let-7b increase following IFN treatment in human hepatocyte HuH7 cells inhibited HCV translation and replication by targeting IGF2BP1 [89, 137]. This implies that let-7b could exert anti-HCV activity via targeting host immune factors and could be used as an anti-HCV therapy and diagnostic test [141]. Recently, Yeh et al. [142] reported that alterations in the expression of let-7b are involved in the progression of HCV infection. Firstly, let-7b can repress the replication of the HCV genome by direct targeting of inhibitors of type 1 IFN signaling. Secondly, let-7b can inhibit the expression of SOCS1 (suppressor of cytokine signaling 1) protein, which acts as a inhibitor of JAK/STAT signaling, leading to increased phosphorylation of STAT1-Y701, thereby increasing the expression of downstream interferon-stimulated genes (ISGs). Let-7 was shown to promote the expression of IFN-β by activation of retinoic acid-inducible gene I (RIG-I) signaling, in addition to direct targeting of the autophagy protein ATG12, and the NF-κB signaling regulator IκB kinase alpha (IKKα) transcripts. This reduced the interaction between RIG-I and the ATG5-ATG12 complex, resulting in increased IFN levels, which in turn activated JAK/STAT signaling. Therefore, the authors concluded that let-7b affected IFN expression by two different signaling pathways [142, 143]. Exploring the regulation of IFN signaling by cellular miRNAs at early stages of HCV infection could clarify the mechanisms underlying primary immune defenses against several types of RNA viruses.

### Herpesviruses (EBV and KSHV)

Herpesviruses are a large family of DNA viruses. These viruses can establish lifelong steady-state infections because of their ability to switch between latent (nonproductive) and lytic stages of infection. To date, eight human herpesviruses have been identified, which are classified into three subfamilies: (i) Alphaherpesvirinae including varicella-zoster virus (VZV), human herpes simplex virus type-1 (HSV-1) and type 2 (HSV-2); (ii) Betaherpesvirinae comprising human herpesvirus type 6 (HHV-6) and type 7 (HHV-7), and cytomegalovirus (CMV); (iii) Gammaherpesvirinae including KSHV and EBV [144, 145]. A variety of diseases ranging from cutaneous lesions to several types of cancer can arise following infection with human herpesviruses. The biological life cycle of herpesviruses comprises two major types of replication: latent and lytic replication. The genes that are expressed during the latency period are required for maintenance of the virus genome in an episomal state, while avoiding damage to the host cells. Therefore, this phase allows the viruses to escape the immune responses of infected hosts and establish a persistent infection [146]. Moreover, some coexisting conditions such as immunosuppression allow herpesviruses to switch the life cycle from latent state to lytic infection, leading to viral gene expression and generation of multiple virions [144, 146].

**Fig. 2:**
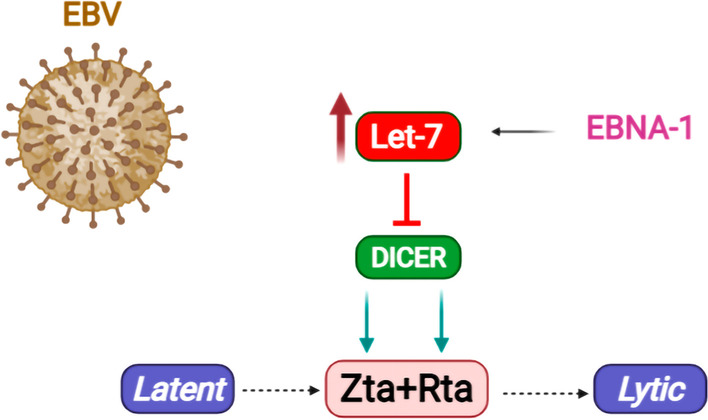
Hsa-let-7b promotes the latency phase of EBV infection by downregulating Dicer

Epstein–Barr virus (EBV) infects the majority of individuals across the world, and has been linked with a number of cancer types, such as gastric carcinoma (GC), B-cell lymphomas (Burkitt and classical Hodgkin), and nasopharyngeal carcinoma (NPC) [147]. The EBV life cycle comprises both latent and lytic infection states in B lymphocytes and epithelial cells. Although EBV remains mostly in the latent phase within B cells, it sometimes switches to the lytic phase to increase cell-to-cell spread. Furthermore, EBV lytic reactivation in the oropharyngeal epithelial cells is required for the generation of infectious viral particles that can be transmitted from host to host. The EBV lytic cycle starts with the expression of the BZLF1 (or Zta) gene, followed by BRLF1 (or Rta) expression. Together these encoded proteins initiate a cascade of subsequent lytic gene expression enabling the biogenesis of linear EBV genomes to be packaged within virions [148]. During the latent phase of infection, a small subset of EBV proteins are expressed and the infected cells become immortalized. The immortalized cells are able to express Epstein–Barr nuclear antigen 1 (EBNA1) during the latent infection [149]. EBNA1 is also the only EBV protein that is crucial for viral replication, and segregation of the viral episomal genomes to maintain a stable copy number of the viral genomes [150, 151]. EBNA1 silencing in GC cell lines was shown to promote EBV reactivation [152]. In 2014, Mansouri et al. [153] observed that several members of let-7 miRNA family (e.g., let-7a) could function as repressors of EBV reactivation in EBV-positive GC and NPC cells. They showed that seven of the let-7 family members were upregulated by EBNA1. EBNA1 overexpression was demonstrated to increase let-7a expression levels in several cell lines, and silencing of EBNA1 reduced let-7a levels. This was proposed to be the mechanism through which EBNA1 could upregulate let-7a expression and inhibit EBV reactivation. Treatment of EBV-expressing cancer cells with a let-7a mimic reduced the percentage of reactivated cells (either via spontaneous reactivation or after chemical treatment with 12-*O*-tetradecanoylphorbol-13-acetate (TPA) and sodium butyrate), and the use of a let-7 sponge reversed these effects. This finding showed that EBNA1 may enhance latent infection probably via induction of let-7 miRNA. Furthermore, it was found out that Dicer was a downstream target for let-7a and EBNA1, suggesting that high Dicer levels may promote EBV reactivation [153]. The results suggested that host let-7 induced the EBV latent state via negative regulation of Dicer (Fig. 2). The precise mechanism by which increased Dicer levels promote EBV reactivation remains to be explored.

KSHV is a gammaherpesvirus that causes Kaposi’s sarcoma (KS), a malignancy derived from virus-infected endothelial cells in the lining of blood and lymph vessels. KSHV also has been associated with primary effusion lymphoma (PEL) and multicentric Castleman’s disease (MCD). Similar to other herpesviruses, KSHV can enter a latent stage in the viral life cycle limiting the expression of viral proteins and immunosurveillance responses, thus allowing persistent infection. The virus infects endothelial cells and B lymphocytes in blood and lymph vessels, and shows dual latent and lytic phases [154]. KSHV maintains the latent phase in most infected host cells, and then undergoes reactivation to facilitate cell-to-cell transmission [155]. This dual-phase viral replication is critical for persistent KSHV infection and for the emergence of KS lesions [156]. Among the genes expressed during the latent phase, the latency-associated nuclear antigen (LANA) protein, encoded by the ORF73 (open reading frame 73) is the most characteristic [157]. LANA has been shown to have an important function as a regulator of gene expression and cell proliferation [158]. The switch from latent to lytic phase in KSHV is regulated by the ORF50-encoded replication and transcription activator (RTA) protein [159]. RTA is required to trigger the expression of the cascade of lytic genes in a sequential order for viral reactivation [160]. RTA binds directly to the corresponding response elements located on gene promoters or indirectly by the DNA-binding adaptors (AP-1, C/EBP-a, OCT-1, or RBPJ) [161]. RBPJ (recombination signal binding protein for the immunoglobulin kappa J region) is a well-conserved protein sequence with widespread cellular expression that functions as the main effector in the Notch pathway [162]. RBPJ plays a role in gene silencing via acting as an adaptor of transactivators, and also recruits corepressor complexes [163]. When RTA binds to RBPJ, it causes further self-activation triggering a positive feedback loop that consequently leads to lytic reactivation [164, 165]. The RTA–RBPJ binding can also promote the expression of a number of lytic genes (e.g., ORF50) in addition to the latent phase transcript cluster including LANA [161, 164, 166]. Furthermore, the RTA-induced LANA also binds to RBPJ to form a negative feedback loop repressing RTA expression, resulting in maintenance of the latent virus [166]. Therefore, RBPJ acts as an intermediate in both the positive and negative feedback loops leading to a balanced regulation of viral replication, in two distinct phases of the KSHV life cycle. Although RBPJ–LANA binding leads to downregulation of RTA, RBPJ is required for the lytic phase but not for the latency phase [167]. In the latent phase of infection, RBPJ does not bind to the promoters, but during viral reactivation the same promoters show a high degree of binding of RBPJ after RTA induction [168]. Moreover, latent virus-infected cells show relatively lower expression of RBPJ, suggesting that RBPJ may be regulated by KSHV replication.

In one of the studies on the association between KSHV and Kaposi’s sarcoma, Tan et al. [169] observed in 2015 that the let-7a values in the tumor tissue were significantly lower than in healthy controls. They also found that KSHV reactivation was inhibited by let-7a, by repression of the miRNA target gene, mitogen-activated protein 4 kinase 4 (MAP4K4). The 3′-UTR of RBPJ was also shown to possess a conserved let-7a-binding site, suggesting that let-7a directly targets RBPJ. Recently, Qi et al. [170] demonstrated the role of LANA in repression of KSHV lytic replication through the let-7a/RBPJ axis. LANA was shown to induce let-7a expression along with repression of RBPJ. LANA upregulates the let-7a expression at the transcriptional level by enhancing the cellular notch intracellular domain (NICD) and inducting let-7a maturation via repressing both Lin28B and NF-κB. Let-7a is able to downregulate RBPJ expression via direct interaction with the 3′-UTR of the target mRNA. In this study, silencing of RBPJ resulted in a time- and concentration-dependent repression of KSHV lytic reactivation. Overall, the results suggested that let-7a miRNA overexpression and subsequent repression of RBPJ mediated by LANA, leads to the maintenance of latent viral infection within the host cells [170]. Additionally, the lytic phase of KSHV is characterized by the expression of the ORF50 gene, which serves as an activator of the lytic phase enabling virus replication, virion assembly, and release of the viral progeny. Another KSHV gene, ORF72 encodes a viral homolog of cyclin D, which is not required for transformation of human T lymphocytes [171]. Zhang et al. [172] assessed the effect of let-7 silencing on KSHV lytic replication. The results showed an increase in gene copy number and mRNA transcripts of both ORF50 and ORF72 genes in response to let-7 silencing. Besides, let-7 silencing was shown to increase the protein levels of MAP4K4, COX-2, and phospho-ERK1/2, while the levels of phospho-JNK and phospho-p38 were unchanged. These results demonstrated that silencing of let-7 miRNA could activate KSHV replication, probably through overexpression of MAP4K4 and its downstream mediators, including MMP-13, COX-2, and ERK1/2 phosphorylation, eventually leading to KS development [172].

### HPV

Human papillomavirus (HPV) is a member of the family of Papillomaviridae with a broad range of hosts including humans and other animals [173, 174]. Over 200 types of HPV have been identified to cause papillomatosis infections, grouped into high-risk or low-risk strains. To date, 20 high-risk types, including HPV-16, 18, and 45, have been identified as causative agents of several human malignancies, including cervical intraepithelial neoplasia and oropharyngeal and anogenital cancers. It has been proven that HPV-16 and 18 are the major cause of cervical cancer worldwide, accounting for 62.6% and 15.7% of total cases, respectively [175, 176]. The circular genome of HPV encodes six early (E1, E2, E4, E5, E6, E7) and two late (L1, L2) proteins. It has been shown that two oncoproteins, namely, E6 and E7, are directly involved in HPV-induced carcinogenesis [173, 175, 177, 178]. Although high-risk HPV strains are the main cause of cervical cancer, most HPV infections are cleared spontaneously in immunocompetent individuals, and only a minority of HPV-infected women will develop cervical cancer [179]. Therefore, additional factors must contribute to cervical malignant progression.

**Fig. 3:**
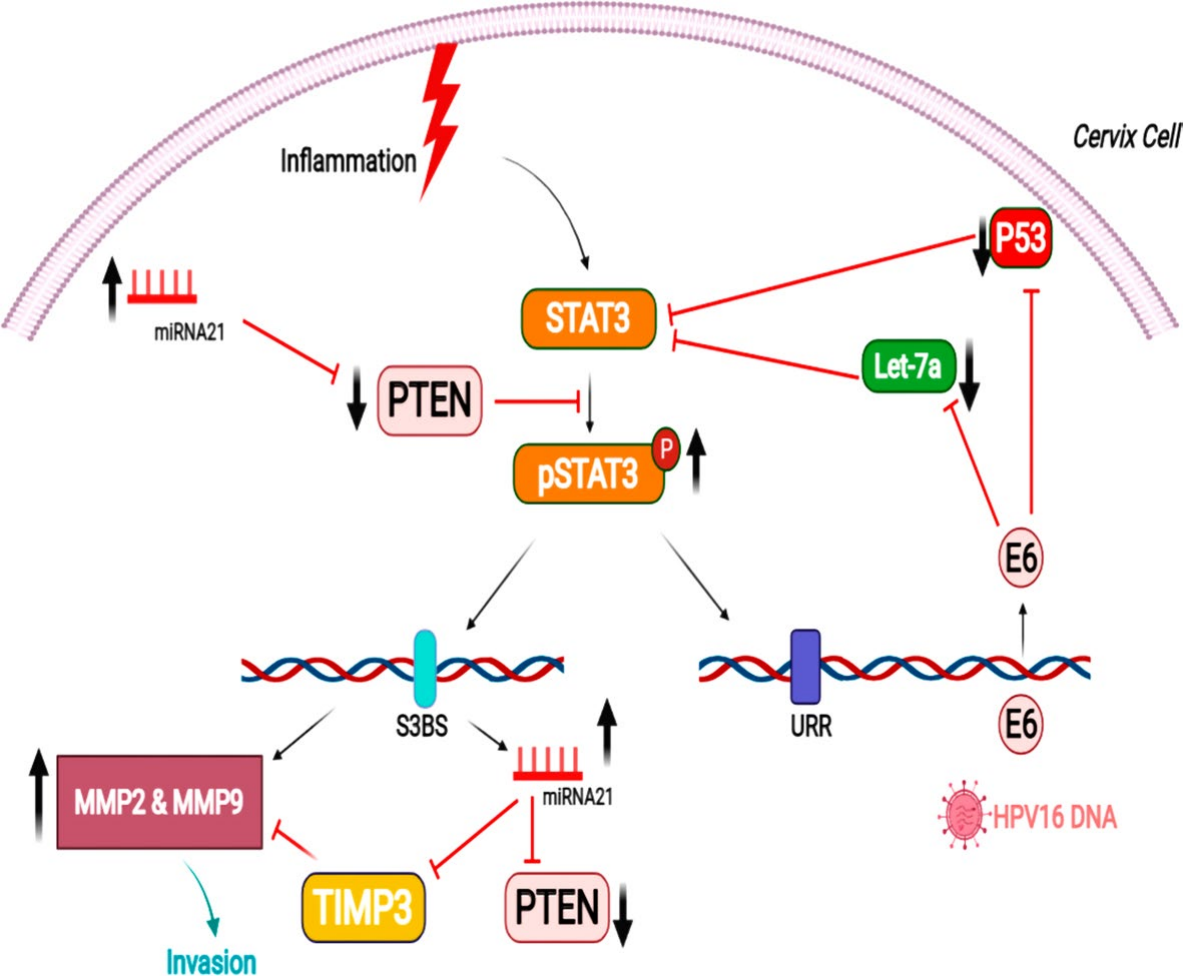
Interaction of HPVE6–miRNA–STAT3 during cervical carcinogenesis. URR, upstream regulatory region; ↑, upregulation; ↓, downregulation; S3BS, STAT3 binding site

Recent evidence has implicated STAT3 in cervical carcinogenesis, which shows elevated expression correlated with the disease stage in HPV-16-positive lesions of the cervix [180–182]. Increased STAT3 activity has been reported in epithelial transformation, and plays a role in HPV-16-associated cervical carcinogenesis [183]. Thus, the increased activation of the STAT3 pathway is a crucial cellular process linking chronic inflammation to cervical cancer development [184]. However, the cellular mechanisms leading to constitutive activation of the STAT3 pathway and alterations in downstream cellular targets remain largely unclear, and need further study for potential therapeutic targeting. Although several molecules have been identified that can positively or negatively modulate the activity and expression of STAT3, recent studies have highlighted the involvement of miRNAs in governing STAT3 expression and its downstream targets [185]. It has been reported that several miRNAs are aberrantly expressed in HPV-infected cervical cancer cells [186–188]. Among the potentially oncogenic miRNAs that could affect the STAT3 pathway, Shishodia and his colleagues investigated the role of two miRNAs, miR-21 and let-7a [189]. The investigation of the miRNA targets showed that let-7a could act as a key regulator of STAT3 expression [124]. Let-7a was found to be often downregulated in cancer cells as a result of chromosomal deletion [190, 191]. Aberrant expression of Let-7a negatively regulates STAT3 transcription via indirect inhibition of IL-6, which is a key cytokine associated with several malignancies, and is also a positive regulator of STAT3 expression [184]. Furthermore, let-7a was observed to directly target STAT3 [124]. Later, Shishodia et al. reported a functional association between the expression levels of miR-21, let-7a, and STAT3 in cervical cancer cells, forming a feedback loop regulated by the oncoprotein E6 [189]. Later, the same researchers [192] measured the levels of miR-21 and let-7a, and investigated their possible correlation with STAT3 in cervical cancer tissues with various grades retrieved from premalignant and malignant lesions in HPV-infected cervical cancer patients. Their results showed that miR-21 was significantly overexpressed. while let-7a was downregulated in cervical cancer tissues. Additionally, miR-21 was directly correlated with the STAT3/ pSTAT3 expression levels, while let-7a demonstrated a reverse correlation in HPV-infected cervical cancer lesions. This reciprocal relationship was not evident for let-7a, especially in precancerous lesions. In HPV-infected lesions, expression levels of miR-21 were correlated with the oncoprotein E6 levels. Contrarily, the let-7a levels were lower in E6-overexpressing lesions, which was consistent with the upregulation of STAT3 mRNA. Unlike miR-21, let-7a showed increased expression in tissue samples from premalignant lesions, compared with higher-grade lesions where let-F7a was downregulated or absent. High expression of let-7a in high-grade squamous intraepithelial lesions (HSIL) but absence in malignant lesions suggests that a major genetic or epigenetic change is involved in malignant switching, which is also required to maintain high levels of STAT3 in malignant cells. More investigations are required to understand the role of let-7a in malignant switching [192]. However, oncoprotein E6 could directly or indirectly via mediators affect the let-7a levels. A negative correlation between let-7a and E6 levels has been found in various clinical samples, while let-7a was also found to inhibit E6 expression in cervical cancer cell lines [189, 192]. Additionally, several studies have shown that silencing of E6 leads to lower STAT3 expression at both transcriptional and translational levels [182, 183, 189]. Moreover, temporary transfection with let-7a mimics (or its precursors with biological function) has been shown to directly and effectively repress STAT3 [189]. Overall, Shishodia et al. hypothesized that in cervical cancer cells there is a strong correlation between lower let-7a expression and increased expression and activation of STAT3, in high-risk HPV-16 infection with higher levels of E6 oncoprotein (Fig. 3). Therefore, miR-21, let-7a, and STAT3 could be used as a potential biomarker signature to discriminate dangerous pre-invasive and malignant cervical lesions, suggesting that STAT3 inhibitors could be tested as a therapy.

## Viral respiratory diseases

Respiratory viruses are the most prevalent cause of human infections, with considerable global morbidity and mortality. Respiratory viral infections account for a high economic burden, leading to a large number of people being absent from school and work and many referrals for medical care, and can also exacerbate underlying chronic respiratory diseases, such as chronic obstructive pulmonary disease (COPD) and asthma [193, 194]. The most common viruses causing respiratory infections include orthomyxoviruses, coronaviruses, adenoviruses, paramyxoviruses, picornaviruses, human bocavirus, and human herpesviruses [195]. Although there are some effective vaccines for respiratory viral infections, they may be difficult to access, and there are only a limited number of antiviral medicines for influenza (oseltamivir and zanamivir); however, there are no clinically effective drugs for the most prevalent viral respiratory diseases [196]. Novel antiviral treatments for prophylaxis and therapy of respiratory viral diseases are required according to the World Health Organization (WHO) Battle against Respiratory Viruses (BRaVe) guideline plan [197].

### Influenza

Influenza virus (IV) is a member of the Orthomyxoviridae family, which are enveloped viruses with single-stranded, negative-sense, segmented RNA genomes [198]. IVs are divided into three genera, A, B, and C. IV genus A is a common cause of respiratory viral disease in humans and can lead to worldwide pandemics, while IV genus B can causing epidemics (but not pandemics), and IV genus C only causes mild respiratory diseases [199, 200].

A number of cellular miRNAs have been reported to show altered expression during IV infections. miRNAs can control translation of viral mRNAs and also their replication either directly or via mediators, thus affecting viral pathogenesis. From the viewpoint of evolution, it is reasonable to hypothesize that IV employs cellular miRNAs to promote its life cycle because it is an obligate intracellular virus [201]. Let-7 is an important regulator of gene expression in epithelial cells [202], and like the miR-200 family, repression of let-7 causes epithelial features to disappear and fibrotic gene expression is increased [203, 204]. Transient miRNA expression has been shown to be altered after IV infection, as revealed by high-throughput microarray assays and deep sequencing techniques. Studies have reported that the expression levels of let-7 family members, miR-29, and miR-30 are all significantly downregulated post-IV infection [205–207]. In another study by Song et al., microarray analysis showed that hsa-let-7g expression was significantly lower in peripheral blood mononuclear cells (PBMCs) obtained from profoundly ill patients diagnosed with H1N1 respiratory infection compared with healthy individuals [208] (Table 1).

Normally, a target mRNA is destroyed when it has a fully matched complementary sequence to the miRNA, while translational repression occurs when the sequences of both RNAs are imperfectly complementary. For instance, Ma et al. [209] reported that several miRNAs showed aberrant expression in IV-positive A549 human lung epithelial cells using high-throughput miRNA microarrays. Among these miRNAs, miR-let-7c in particular showed the highest expression in the lung epithelial cells. Also, they showed that miR-let-7c possessed a fully matching complementary sequence to the 3′-UTR of the H1N1 M1 gene, and could downregulate M1 expression at both the (+) cRNA and protein levels. These results suggested that the let-7c binding to the 3′-UTR of M1 could be used for protection and therapy of IV infections [209].

In a pioneering work, Landgraf et al. produced a comprehensive atlas containing data on the expression of known miRNAs in a large variety of cells and tissues in both mice and humans. They showed that, although most cellular miRNAs are extensively expressed, a number are exclusively expressed in a specific cell type, lineage, or tissue [210]. However, various miRNAs with specific tissue or host expression could affect viral gene expression to different extents. The level of reduction in gene expression is particularly important when aiming to develop a miRNA-attenuated vaccine. IVs used in the production of vaccines provide high titers of virus when grown in chicken egg embryos. To produce IV vaccines in chicken eggs that were attenuated in mammalian cells, Perez et al. [211] evaluated the miRNAs in avian and mammalian cells, and discovered several that were found in mammalian cells but not in eggs. They chose miR-93 to develop an IV vaccine that could replicate in eggs while remaining attenuated in mice [211]. This vaccine demonstrated strong immunity against lethal IV strains in mice. Another potential application of these miRNAs with species-specific attenuation is to increase safety when handling potentially dangerous viruses during molecular research interventions. However, the Perez et al. study had several drawbacks, including that the miR-93 level in lung tissue is rather low, and also the targeted protein in IV is a structural protein and is not involved in viral replication [212]. For this reason, Shen et al. [212] inserted a miRNA recognition element (MRE) for let-7b into the polymerase gene PB1 of IV to develop a modified H1N1 virus with specific replication in the lungs. This was because let-7b is highly expressed in bronchial epithelial cells, and PB1 is an RNA-dependent RNA polymerase that plays a crucial role in viral replication. Insertion of the let-7b-MRE caused the engineered H1N1 to become susceptible to miR-let-7b targeting, showing that viral pathogenicity could be attenuated through incorporation of a replication-restrictive element. Recently, let-7b target sequences were incorporated by Feng et al. into the PB1 of pandemic H1N1 virus identified in 2009 (A/Nanjing/NJU-108/2009) to develop an engineered virus (miRT-H1N1) [213], and the antiviral protection was evaluated after immunization in BALB/c mice. The results showed that the miRT-H1N1 virus was attenuated in infected mice, whereas it maintained wild-type virulence in chicken embryos. Also, the vaccinated mice exhibited strong immunity against lethal A/Nanjing/NJU-108/2009 infection. They suggested that an IV containing an MRE is weakened in vivo and could be used to design a live attenuated vaccine. Overall, miRNAs with specific expression in a certain type of cell or species (e.g., let-7b) could be used to modify pathogenic viral replication to develop live attenuated vaccines. These findings suggest that let-7 miRNA could provide a novel potential biomarker for influenza infection.

### SARS CoV-2

Severe acute respiratory syndrome coronavirus 2 (SARS-CoV-2) is a member of the family Coronaviridae, subfamily Coronavirinae, genus *Betacoronavirus*, and was identified as the causative agent of the novel coronavirus disease in 2019 (COVID-19) [214]. This virus was initially identified in a number of pneumonia cases in Wuhan, China in late 2019 [215–217]. Now, the pandemic of SARS-CoV-2 has spread around the globe, and has led to the death of a constantly increasing number of people, now over 4.7 million. Over 231 million coronavirus cases have been diagnosed since composing this article [218]. Novel approaches and comprehensively available vaccines are required to control this lethal disease and similar pandemic outbreaks.

Xie et al. [219] hypothesized that let-7 could inhibit COVID-19 by targeting the causative virus. They conducted a series of computational assays to detect putative let-7 target sites located on the SARS-CoV-2 genome, and discovered two sites with complementary sequences matching the seed region of let-7-3p, located in the coding regions of viral S and M proteins. Functional assessment showed that a number of let-7 family members (let-7d, e, f, g, i) and miR-98 could inhibit expression of the S protein, whereas others (let-7b, c, g, i) and miR-98 could inhibit M protein expression. Additionally, some reports showed that let-7a and let-7c were able to inhibit IL-6 expression, which is an inflammatory cytokine highly expressed in COVID-19 patients [184]. They speculated that higher let-7 expression could lower some inflammatory cytokines and chemokines, but not IL-6. This effect could potentially benefit patients by controlling the virus-induced cytokine storm. Xie et al. [219] reported that ectopic expression of let-7a or let-7c decreased IL-6 mRNA, in addition to other SARS-CoV-2-associated inflammatory mediators, such as CCL2, TNF-α, IL-1β, IL-8, VEGFα, and GM-CSF. They also showed that let-7-5p could upregulate IL-6, IL-1β, IL-8, TNF-α, and GM-CSF, while sponging of let-7-3p enhanced GM-CSF, IL-8, TNF-α, and CCL2 expression in THP1 leukemic cell cells that had been treated with lipopolysaccharide (LPS). These findings indicated the potential of let-7 miRNA to suppress damaging inflammatory responses [219].

Approaches to control SARS-CoV-2 infection include targeting the replication machinery of the virus, inhibiting virus binding to host receptors, and blocking the function of viral proteins [220]. It has been reported that cellular miRNAs can inhibit SARS-CoV-2 gene expression at the translational level via binding to 3′-UTR of the targeted viral genes. This can block the host cell receptors and alter viral structural and functional proteins, but does not affect gene expression in the host cells. Demirci et al. identified some potential viral targets that could be affected by human miRNAs. They discovered that several SARS-CoV-2 genes, including ORF6, were affected by multiple host miRNAs. For instance, let-7c-5p was found to target the viral ORF1ab, and inhibit its replication [221]. Sardar et al. identified six human miRNAs that recognized SARS-CoV-2 proteins: let-7a and miR-101 (targeting nonstructural proteins), miR-126 and miR-378 (targeting the N region), and miR-23b (targeting the S region) [222]. Moreover, let-7 could improve immunity against SARS-CoV-2, because the virus suppresses immune responses partly by suppressing host miRNAs.

### Respiratory syncytial virus

Respiratory syncytial virus (RSV) is a common pathogenic virus causing pediatric and geriatric infections, with a considerable number of hospitalizations, clinic visits, and > 14, 000 deaths globally each year. RSV belongs to the genus *Paramyxovirus* with a negative-sense single-stranded RNA (ssRNA) genome, 15 kb in length encoding 11 proteins, that is, NS1, NS2, M, N, P, L, F, G, SH, M2-1, and M2-2. Although, over the past 60 years, scientists have attempted to design an RSV vaccine, these efforts have not yet provided effective agents for prophylaxis and treatment of RSV. These failures are largely due to our lack of knowledge about the host–virus interactions [223]. Some studies have tried to explore the host-to-virus interface to develop a vaccine against RSV. miRNAs have been found to play a role in virus–host interactions, and could be useful in therapeutic and prophylactic strategies. For instance, Bakre et al. [223] reported that RSV-infected-A549 human alveolar epithelial cells showed overexpression of five miRNAs, that is, miR-24, miR-26b, miR-337-3p, let-7f, and miR-520a-5p. They also showed downregulation of two others, miR-595 and miR-198. The virus G protein can modify the expression of some inflammatory factors, whereas it suppressed type I IFN via induction of suppressor of cytokine signaling 1 (SOCS1) and SOCS3 [224, 225]. They infected A549 human alveolar epithelial cells with recombinant RSV (6340WT) or a recombinant virus with mutated G gene (RSVΔG) to investigate the effects of viral G protein on miRNA expression, particularly let-7, and found that RSV G protein increased let-7 expression [223]. They also demonstrated that let-7f controlled the expression of SOCS3 and CCL7/MCP3, which are known to play a role in the host inflammatory response. Previous studies have reported that the G protein inhibited the release of chemokines by bronchoalveolar leukocytes in response to RSV infection [224], but increased the expression of IL-8 [224]. Let-7f was shown to control the expression of early flowering 4 (ELF4), which in turn increases IL-8 expression [226]. The expression of RSV G protein was correlated with let-7f repression, and subsequently reduced the expression of IL-8. It has been reported that some amino acid residues located on the cysteine loop region of RSV G protein contribute to its modulatory effect on IFN-λ expression, and can suppress several miRNAs, including let-7f [227]. Taken together, RSV represses the expression of let-7 miRNA family members to escape from the the host antiviral defense, partly via the crucial RSV G protein.

## Let-7 and HIV

The human immunodeficiency virus 1 (HIV-1) is an enveloped RNA virus with a genome composed of two identical single-stranded sequences. HIV-1 is a member of the *Lentivirus* genus, Retroviridae family, and Orthoretrovirinae subfamily, and is the causative agent of acquired immunodeficiency syndrome (AIDS). HIV-1 initially binds to and replicates inside CD4^+^ T lymphocytes, as well as monocytes and macrophages. The three stages of HIV-1 infection are the acute phase, chronic phase, and appearance of AIDS. HIV-1 infection may take an average time of 10 years before the development of AIDS [228].

Only low levels of HIV markers (e.g., HIV-1 RNA and p24) can be identified immediately after HIV-1 infection, and during the early viral infection stage these markers may not be detected at all. Therefore, the early stage has been called the “window period” or “eclipse stage” [229]. Typically, HIV-1 markers could be detected 10 days after viral infection, when the viral RNA can be identified using nucleic acid test (NAT) assays [230–232]. After that, 15–22 days following infection, HIV-1 p24 antigen becomes detectable. Acute HIV infection is defined as the interval between virus acquisition and development of seroconversion. The acute phase is highly infectious with peak levels of viral load in the circulation, occasionally reaching > 1 × 10^7^copies/mL [233]. Additionally, the viral load accompanied with the absence of neutralizing antibodies produces a high HIV transmission rate [234, 235]. Subsequently, immunoglobulins (IgM and IgG) against HIV-1 appear, which are easily detected using immunoassays [236, 237]. High rates of false-negative results have been reported in the window period of HIV-1 infection [238]. Precise diagnostic approaches for detection of HIV are needed to minimize the risk of HIV transmission from infected individuals. Individuals in the acute phase of HIV infection mostly do not know they have been infected, and may continue to carry out high-risk behavior, thus increasing transmission rates. Accordingly, early screening of HIV-1-infected individuals could help to prevent viral transmission [239]. Therefore, host-associated prognostic and predictive biomarkers are needed in addition to the currently available diagnostic strategies. Identification and validation of cellular miRNAs as potential biomarkers may help us achieve this goal. Recently Biswas et al. [240] assessed the miRNA expression profiles in early-stage HIV-1-infected subjects, in an attempt to create a miRNA-based approach for prediction. They found that four miRNAs showed differential expression in HIV-1-infected individuals (miR-20b-5p, miR-16-5p, miR-223-3p, and miR-195-5p) and could be used to distinguish early infected subjects from non-infected subjects, with high diagnostic power [AUC 1.000 (95% CI 1.00–1.00), sensitivity 100%, and specificity 100%]. In addition, to diagnose the HIV-1-infected individuals within the window period, they created a different four-miRNA-based panel (let-7g-3p, miR-206, miR-16-5p, and miR-181c-3p) also with high diagnostic power [AUC 0.999 (95% CI 0.995–1.000), sensitivity 100%, and specificity 95·8%]. Furthermore, the use of let-7g-3p alone could distinguish early HIV-1-infected subjects from healthy subjects [AUC 0.91 (95% CI 0.81–1.0)] [240] (Table 2).

**Table 2:** The role of let-7 family members in viral infections

Virus	Let-7 member	Expression	Target	Model	Note	Refs.
HBV	Let-7g	Up	preS2	In vitro (Hep38.7)	Anti-HBV activity Decreased level of HBV cccDNA and HBV replication	[115]
HBV	Let-7a	Down	c-myc CCR7 K-RAS	Human (tissue samples of HCC, *n* = 20) /in vitro (Huh-7)	HBV mRNAs (pre-C/C, pre-S, and S) promoted the progression of HCC by decreasing the expression level of let-7a. mRNAs de-repressed let-7a targets, including c-myc, K-RAS, and CCR7	[116]
HBV	Let-7a	Down	STAT3	In vitro (HBx-HepG2)	HBX protein enhanced cell proliferation by decreasing the expression level of let-7a	[124]
HBV	Let-7i	Down	CD59	Human (HCC tissues samples)/ in vitro (HBx-HepG2, HBx-L-O2)	HBx increased CD59 expression through (probable) downregulation of let-7i levels	[125]
HCV	Let-7c	Up	Bach1	In vitro (Ava.5/ JFH1-Huh7)	Anti-HCV activity let‐7c suppressed HCV replication by targeting Bach1	[138]
HCV	Let-7b	Up	SOCS1 IKKα ATG12	In vitro (Huh7)	Anti-HCV activity let-7b inhibited HCV by enhancing JAK/STAT and RIG-I signaling pathways during the early stage of HCV infection	[142]
HCV	Let-7b	Up	IGF2BP1	In vitro (IFN-α and IL-28B treated Huh7, Huh7.5.1)	Anti-HCV activity by targeting IGF2BP1 Let-7 s reduced HCV replication and translation	[137]
HCV (genotype 1b, 2a)	Let-7g	Up	5′-UTR of HCV genome	In vitro (PEG-IFN/RBV-treated Ava.5- Huh7, JFH1-Huh7.5.1)	Anti-HCV activity Let-7g cooperated with interferon/ribavirin to repress hepatitis C virus replication through p38/AP-1 signaling	[287]
HCV (genotype 1b)	Let-7b	Up	NS5B 5′UTR	In vitro (Huh-7)	Anti HCV activity Let-7b suppressed replication and translation of HCV by targeting NS5B and the 5′-UTR region of HCV genome	[135]
HCV (genotype 1b)	Let-7a	Down	CLDN1 CDH1	Human (tissue samples of chronic HCV)/in vitro (Huh7.5.1, PHH)	Let-7a was significantly downregulated by HCV Let-7a and 7b restricted multiple steps of the HCV life cycle, including entry, translation, and RNA replication	[139]
HIV-1	Let-7c	Up	CDKN1A, at the RNA and protein (p21) levels	In vitro (T lymphocytes, HeLa-CCR5)	Let-7c was upregulated on the first day after HIV infection and downregulated at later timepoints. Upregulation of has-let-7c levels resulted in enhanced HIV replication	[256]
HIV-1	Let-7i	Down	IL-2 promoter TATA-box region	In vitro (CD4^+^ T cell)	HIV-1 infection attenuated the expression of let-7i and promoted the activity of IL-2	[250]
HIV-1	Let-7b Let-7c Let-7f	Down	IL-10	In vitro (HUT78)	Let-7 decreased IL-10 levels Downregulation of let-7 miRNAs by HIV infection may result in an increase in IL-10 secretion from CD4^+^ T cells, providing the virus with a survival advantage	[123]
HHV-8	Let-7a	Up	RBPJ	In vitro (293 T)	LANA protein repressed lytic replication of HHV-8 by upregulating let-7a expression and promoting notch intracellular domain (NICD) and decreasing LIN28B and NF-κB	[170]
HHV-8	Let-7a Let-7d Let-7e Let-7i	Down	MAP4K4	Human (tissue samples, *n* = 4) /in vitro (293 T)	Anti-KSHV activity Let-7a inhibited replication of KSHV by targeting MAP4K4 signaling pathways	[169]
HPV-16 and HPV-18	Let-7a	Down	STAT3	Human (tissue samples of cervical cancer, *n* = 53)/in vitro (CaSki, SiHa, HeLa)	E6 increased expression level of STAT3 by downregulation of let-7a	[192]
EBV	Let-7a-5p Let-7b-5p Let-7d Let-7e-5p Let-7f-5p Let-7g-5p	Up	BZLF1 Dicer	In vitro (HONE1, CNE2Z)	EBV EBNA1 promoted EBV latency by inducing the expression of let-7	[153]
SARS-CoV-2	Let7-d-3p Let7-e-3p Let7-f-3p Let7g-3p Let7-i-3p	Up	S	In vitro	Has-let-7 repressed SARS-CoV-2 replication by targeting S gene of virus	[219]
SARS-CoV-2	Let7-b-3p Let7-c-3p let7g-3p Let7-i-3p	Up	M	In vitro	let-7c-5p can target the ORF1ab SARS-CoV-2 to inhibit its replication	[219]
H1N1	Let-7c	Up	M1	In vitro (A549)	Inhibited virus replication	[209]
H7N9 Avian	Let-7e	Down	IL-6	In vitro (THP‑1)	The expression of pro-inflammatory factors IL‑6, IL‑1α, and IL‑1β was promoted through the effect of HA protein on let-7e expression	[320]
RSV	Let-7f	Up	SOCS3 CCND1 ELF4 DYRK2 CCL7	In vitro (A549)	G protein stimulated expression of let-7f, to promote virus replication	[223]
RSV	Let-7f	Down	IFN λ	In vitro (Calu-3)	G protein led to escape from interferon response by altering expression of let-7f	[227]
DENV-2	Let-7e	Up	SOCS3	In vitro (PBMC)	Enhanced the level of pro-inflammatory cytokines during infection	[321]
DENV-2	Let-7a	Up	NS1	In vitro (Huh-7)	Decreased NS1 RNA and protein expression, repressed DENV virus replication and pathogenesis	[322]
Enterovirus 71	let‑7c‑ 5p	Up	MAP4K4	In vitro (rhabdomyosarcoma)	Hsa-let-7c-5p promoted enterovirus 71 replication by activating the JNK signaling pathway	[323]
Enterovirus 71	Let-7b	Up	CCND1	In vitro (SH-SY5Y)	Increased cell apoptosis	[324]
Enterovirus 71	Let-7a	Up	VP2 5′UTR	In vitro (SK-N-SH, RD)	Suppressed virus replication and decreased viral load	[325]
Pestivirus	Let-7a Let-7b	Up	3′-UTR	In vitro (MDBK)	Promoted virus replication, translation, and RNA stability	[326]
Porcine reproductive and respiratory syndrome virus	Let-7f-5p	Down	MYH9	In vitro (HEK293FT)	Repressed virus replication	[327]

Interleukin 10 (IL-10) is a multifunctional cytokine that is expressed in a most immune cells [241]. Plasma IL-10 levels have been found to be higher in subjects with HIV-1 infection, and this is thought to contribute to the poor antiviral immune response by CTLs. The levels of IL-10 expression can be controlled at several levels, including the epigenetic level via different signaling pathways, at the transcriptional level via transcription factors, and the post-transcriptional level mediated by miRNAs [242, 243]. Swaminathan et al. [123] showed that let-7 could repress IL-10 expression at the post-transcriptional level. They found that IL-10 was highly upregulated in HUT78 T cells, and proposed that let-7 overexpression decreased IL-10, because silencing of let-7 miRNA led to a significant increase in IL-10 levels. HIV-1 infected HUT78 cells showed lower let-7 levels accompanied by increased IL-10 levels, suggesting that the decreased let-7 level may be involved in the increased IL-10 expression that was seen in HIV-1 infection. Also, they found reduced let-7 levels in primary CD4^+^ T cells retrieved from blood samples of subjects with HIV-1 infection, compared with non-infected controls, suggesting that the altered miRNA levels could be linked to the increased IL-10 expression in HIV patients. They proposed that dysregulation of the let-7/IL-10 axis could result in the abnormal CTL function seen in HIV-1-infected individuals [123].

Interleukin-2 (IL-2) is an essential cytokine that regulates the cell number, differentiation state, and death of most T cells. IL-2 is mainly released from activated Th cells, and is controlled by several transcription factors, chromatin remodeling agents, and the CD28 co-stimulation pathway [244, 245]. In a number of studies, HIV-1 infection has been shown to suppress IL-2 expression in Th cells in vitro [246–249]. Recently, Zhang et al. [250] reported that let-7i induced gene expression in Th cells by binding to the TATA-box of the IL-2 promoter, and promoting the assembly of pre-initiation complexes, which are required for mRNA transcription. They observed that HIV-1 infection results in lower levels of mature let-7i, as well as its precursor and primary forms. Additionally, studies have shown that the function of the let-7i promoter is reduced in Th cells following HIV-1 infection. As a result, they suggested that viral infection results in suppression of the let-7i/IL-2 axis contributing to Th cell death. This was a newly described mechanism for HIV-1-induced Th cell death, because IL-2 cytokine can enhance the survival of activated T cells [250]. Furthermore, because IL-2 is known to regulate the balance of the immune system, the let-7i/IL-2 pathway could be responsible for other immune deficiencies seen after HIV-1 infection (e.g., T-cell functional unresponsiveness) [251–253].

A number of miRNAs have been proposed to facilitate HIV-1 infection by suppressing proteins involved in the host antiviral response. For instance, p21 protein inhibits HIV-1 gene expression by blockading the CDK9 transcriptional elongation factor, while the TASK1 K^+^-channel protein inhibits the Vpu-mediated increase in HIV-1 release [254, 255]. Farberov et al. [256] showed that targeted reduction of TASK1 and p21 expression by miR-124a or miR-34a-5p and Let-7c, respectively, could increase HIV-1 replication in HeLa-CCR5 and JLTRG-R5 cells [256].

## Extracellular vesicles loaded with let-7 in viral infections

Extracellular vesicles (EV) are membranous structures that have been classified on the basis of vesicle size, function, or biogenesis process. According to the International Society of EVs, they can be divided into microvesicles (MVs), exosomes, and apoptotic bodies (ABs) [10]. Due to the potential capacity of EVs to transport macromolecules, such as proteins and RNA transcripts, from source cells to recipient cells, they have gained considerable attention. Exosome-mediated transfer of mRNAs and miRNAs has been shown to alter processes within the recipient cells, such as regulate protein expression, indicating that exosome-derived RNAs can play functional roles. Moreover the RNA profile of EVs from a daughter cell can vary significantly from the parental cell, which means that cells can change the composition and concentration of RNAs in EVs [11]. Some populations of EVs have been discovered with high amounts of miRNAs amounting to 39% of the entire RNA content of EVs, but only 6% of the total cellular vesicles [257]. This suggested that miRNAs can be selectively and purposefully sorted and packaged into EVs.

The EVs that originate from virus-infected cells have been found to be able to transfer proteins, viral genomes, and host factors from donor to adjacent recipient cells, or from infected tissue to other tissues. This may lead to modulation of the host immune response to encourage the establishment of a productive viral infection [258, 259]. Additionally, the transfer of miRNAs through EVs could facilitate virus spread through modulating immune responses [260, 261]. These finding suggested that exosomes are involved in viral replication; however, the precise mechanisms for this, and the effects on immune system defense, are not yet clarified.

Many studies have evaluated the biological function of different miRNAs in HCV infection. miRNAs within the EVs may enable communication between neighboring cells and affect gene expression in the recipient cells [5]. Matsuura et al. [262] designed a study to assess whether circulating miRNAs contained within EVs could predict disease progression in CHC patients compared with the hepatic expression levels, and investigated the mechanism of the association. They performed a large miRNA profiling study in plasma samples from CHC patients using a microarray technique. The authors found that 323 miRNAs were differentially expressed in CHC patients compared with healthy controls; however, only six of these could distinguish mild hepatitis subjects from patients with severe chronic hepatitis. Let-7d-5p, let-7a, let-7c, and miR-122-5p were promising in terms of predicting disease progression in CHC patients. There was an inverse association between plasma let-7 miRNA and histological fibrosis stage and fibrosis-related markers. Let-7 values in EVs performed similarly compared with plasma values to discriminate hepatic fibrosis. Longitudinal evaluation in a total of CHC 60 patients showed that plasma expression of let-7 significantly decreased over time, in agreement with the progression of liver fibrosis determined by sequential biopsies. They concluded that the let-7 miRNA family showed the best association with the progression of liver fibrosis in CHC patients. Later, the same researchers [133] evaluated the correlation between the serum circulating let-7a-5p values and EVs isolated from the serum of 84 Japanese patients diagnosed with CHC and the correlation with hepatic fibrosis severity in paired liver biopsies. They found that serum let-7a-5p values and let-7a-5p in EVs were significantly reduced in liver cirrhosis patients. Additionally, let-7a-5p values significantly correlated with hepatic fibrosis markers and could predict hepatic cirrhosis more accurately than other markers of hepatic fibrosis [133]. They proposed that the lower levels of let-7 could affect liver fibrogenesis after viral infection by triggering the TGF-β signaling pathway [262].

More than 36 million people around the world are living with HIV infection. Among them, about 2.3 million people are also estimated to be coinfected with HCV [263]. Due to the similar routes of viral transmission and similar high-risk behaviors, HIV-infected patients are at higher risk of HCV infection. Hepatic disorders are now the leading cause of morbidity and death in individuals who are infected with both HIV and HCV. These patients are likely to develop advanced hepatic disease with a more rapid progression rate compared with those who only have HCV mono-infection [264]. Moreover, HCV and HIV exploit the same host exosomal machinery to promote infection and evade the immune response, leading to alterations of the small RNA cargos after viral infection. Martínez-González et al. [263] investigated the small RNA cargo profile of EVs extracted from plasma samples of HIV/HCV-coinfected individuals. They reported that three miRNAs had specific expression in the liver, and miR-21-5p, hsa-miR-let-7a-5p, and hsa-miR-122-5p were upregulated in patients who were coinfected with HIV and HCV, suggesting that EV miRNA cargos could provide information on liver disease progression [263].

Microglia are specific types of macrophages with a mesodermal/mesenchymal origin generally located in the CNS, and are critical mediators of inflammation within the CNS (neuroinflammation) [265]. Neuroinflammation is a characteristic sign of Japanese encephalitis virus (JEV) infection. This virus is neurotropic in nature and typically affects young children (1–5 years old), sometimes resulting in lifelong consequences, like neuronal damage, motor disability, and amnesia [266]. During the past years, it has been found that JEV infection can cause dysregulations in the miRNA profile within the brain, and some miRNAs are involved in the regulation of JEV replication and neuroinflammatory responses [267–269]. Nevertheless, the possible role of neurologic cell–cell communication via microglial-derived miRNAs in viral infections has not been explored. Recently, Mukherjee et al. reported that let-7b and let-7a (let-7a/b) miRNAs were upregulated in, and released from, JEV-infected microglial cells incorporated inside EVs [270]. Reportedly, the let -7a/b miRNA could modulate the inflammatory response in microglial cells via activation of the Toll-like receptor 7 (TLR7) signaling pathway [271]; however, the precise role in promoting JEV pathogenesis is unclear. To explore this further, Mukherjee et al. [272] designed a study to clarify the role of let-7a/b in the pathogenesis of JEV. They first evaluated the effect of miRNA-loaded exosomes on primary neurons. They reported that inhibition of TLR7, or addition of let-7a/b, inhibited the JEV-induced activation of the NOTCH pathway, possibly via the NF-κB pathway, and eventually reduced the JEV-induced generation of TNF-α in microglial cells. Additionally, delivery of isolated exosomes released from let-7a/b-overexpressing microglial cells to the brains of healthy mice caused activation of caspases. When neuro2a neuronal cells or primary cortical neurons were incubated with exosomes derived from JEV-infected cells, or with miRNA-overexpressing microglial cells, both induced caspase activation resulting in cell death. Therefore, these findings underline the complex role of let-7a/b involved in JEV pathogenesis. Because let-7a/b can interact with the TLR7 and NOTCH pathways, it can increase TNF-α secretion from microglial cells. Contrarily, exosomes from virus-infected microglia may activate caspases in healthy adjacent neuronal cells, contributing to their death [272] (Table 3).

**Table 3:** Exosomes containing let-7 family members in viral infections

Virus	Let-7 family	Expression	Exosome source	Detection technique of miR	Sample (^n^)	Refs.
HCV	Let-7a-5p Let-7d-5p	Up	Plasma of chronic hepatitis	qRT-PCR	32	[262]
HCV	Let-7c-5p	Down	Plasma of chronic hepatitis	qRT-PCR	32	[262]
HCV	Let-7a-5p	Down	Serum of chronic hepatitis with liver cirrhosis	qRT-PCR	25	[133]
HCV/HEV	Let-7i	Down	Serum of blood donors	qRT-PCR	4	[328]
HIV	Let-7a Let-7d Let-7e Let-7f Let-7g Let-7i	Up	Plasma of heroin abuse	Microarray qRT-PCR	19	[329]
HIV/HCV	Let-7a-5p Let-7b-5p Let-7f-5p	Up	Blood	Sequencing qRT-PCR	4	[263]
Japanese encephalitis virus (JEV)	Let-7a Let-7b	Up	N9 cells	qRT-PCR	–	[270]
Japanese encephalitis virus (JEV)	Let-7g-5p	Up	CSF of acute encephalitis	qRT-PCR	16	[267]
HPV 18	Let-7d-5p	Down	HeLa cells	qRT-PCR	–	[330]
H1N1	Let-7b-5p	Down	BALF of influenza with acute respiratory distress syndrome (ARDS)	NGS	6	[331]
H7N7 Avian	Let-7a	–	A549 cells	Microfluidic microarray platform	–	[310]
H7N7 Avian	Let-7b	–	A549 cells	Microfluidic microarray platform	–	[310]
H7N7 Avian	Let-7c	–	A549 cells	Microfluidic microarray platform	–	[310]
H7N7 Avian	Let-7d	–	A549 cells	Microfluidic microarray platform	–	[310]
H7N7 Avian	Let-7e	–	A549 cells	Microfluidic microarray platform	–	[310]
H7N7 Avian	Let-7f	–	A549 cells	Microfluidic microarray platform	–	[310]
H7N7 Avian	Let-7i	–	A549 cells	Microfluidic microarray platform	–	[310]
H1N1 Swine/H7N7 Avian	Let-7g	–	A549 cells	Microfluidic microarray platform	–	[310]

## Conclusion

We have shown that the let-7 family of miRNAs functions as a regulator of a number of crucial cellular processes. We summarized how let-7 miRNAs can affect viral pathogenesis. Investigators are currently attempting to identify new therapeutic drugs for the treatment of viral diseases and cancer-associated viruses. Recently miRNA-based therapeutic approaches have emerged as promising candidates to meet this goal. The role of let-7 role in some viral infections is well understood, whereas its role in other viral diseases (in particular oncovirus infections) is more complicated and still a matter of debate. It has been shown that let-7 miRNAs are able to reduce the expression levels of several target genes, including MAP4K4, STAT3, IL-10, ORF1ab, SARS-CoV-2, and H1N1 M1 genes. These changes in gene expression may affect the antiviral response of the human immune system. There is evidence that let-7 can be extracted from different human bodily fluids, and the expression values of let-7 are different in virus-infected patients relative to healthy individuals, suggesting its potential to be used as a diagnostic biomarker in clinical settings. Moreover, because let-7 plays such a crucial role in the development of virus and cancer-associated virus infections, it could serve as a valuable target for novel treatments of viral infections.

## Data Availability

Not applicable.
